# Interferon beta induces apoptosis in nasopharyngeal carcinoma cells *via* the TRAIL-signaling pathway

**DOI:** 10.18632/oncotarget.24479

**Published:** 2018-02-12

**Authors:** Anna Makowska, Lora Wahab, Till Braunschweig, Nikiforos-Ioannis Kapetanakis, Christian Vokuhl, Bernd Denecke, Lian Shen, Pierre Busson, Udo Kontny

**Affiliations:** ^1^ Division of Pediatric Hematology, Oncology and Stem Cell Transplantation, Medical Faculty, RWTH Aachen University, Aachen, Germany; ^2^ Institute of Pathology, Medical Faculty, RWTH Aachen University, Aachen, Germany; ^3^ CNRS UMR 8126, Gustave Roussy and Université Paris-Sud/Paris-Saclay, Villejuif, France; ^4^ Institute of Pathology, Kiel Pediatric Tumor Registry, Christian-Albrechts-University, Kiel, Germany; ^5^ IZKF, Medical Faculty, RWTH Aachen University, Aachen, Germany

**Keywords:** nasopharyngeal carcinoma, interferon beta, TRAIL, apoptosis, siRNA

## Abstract

The combination of neoadjuvant chemotherapy, radiochemotherapy, and maintenance therapy with interferon beta (IFNβ) has led to superior results in the treatment of children and adolescents with nasopharyngeal carcinoma (NPC). However, nothing is known about the mechanism of the antitumor activity of IFNβ in NPC. Here, we investigate the role of IFNβ on apoptosis in NPC cells. Six NPC cell lines, one patient-derived NPC xenograft (PDX) and one SV40-transformed nasoepithelial cell line were used. Induction of apoptosis by IFNβ was measured by flow cytometric analysis of subG1-DNA-content, Hoechst 33258 staining and activation of caspase-3. Dissection of death ligand signaling pathways included measuring surface expression of its components by flow cytometry, activation by death ligands and neutralization with specific antibodies and siRNA. IFNβ induced apoptosis at concentrations achievable in humans in five of six NPC cell lines and in PDX cells but not in nasoepithelial cells. Inhibition of caspases-3 and −8 abrogated this effect suggesting IFNβ promoted apoptosis through the extrinsic pathway. IFNβ induced surface expression of TRAIL and TRAIL-R2 and the addition of an anti-TRAIL-antibody or transfection with TRAIL-siRNA blocked IFNβ-induced apoptosis. No induction of TRAIL-expression was noted in the IFNβ-resistant cell line. In conclusion, IFNβ leads to apoptosis in NPC cells in an autocrine way *via* the induction of TRAIL expression and subsequent activation of the TRAIL-signaling pathway. The mechanism described could at least partly explain the clinical benefit of IFNβ in the treatment of NPC. Further studies in a mouse-xenograft model are warranted to substantiate this effect *in vivo*.

## INTRODUCTION

Nasopharyngeal carcinoma (NPC) is a malignant tumor arising from the surface epithelium of the posterior nasopharynx. Etiologic studies have demonstrated that NPC follows a multistep process, in which Epstein-Barr-Virus (EBV), ethnic background and environmental carcinogens seem to play an important role [1–2]. Most cases of NPC in children, adolescents and young adults present as advanced locoregional disease [3–7]. Survival chances for children with locoregional disease range between 80% and 90% and failures are mostly due to the development of distant relapses. In comparison, the survival chances for children with metastatic NPC at diagnosis are less than 10%. Whereas simultaneous radiochemotherapy has been considered as standard therapy for adults with nasopharyngeal carcinoma [8], in children and adolescents, the addition of neoadjuvant chemotherapy has led to superior results [4–7]. The highest survival rates for localized NPC in children and adolescents, with overall survival rates and event-free survival rates >90%, have been achieved with the GPOH-NPC-91 and -NPC-2003 studies, and this with lower radiation dosages compared with other prospective studies in pediatric NPC [5–6]. The GPOH concept is unique by the fact that in addition to neoadjuvant chemotherapy followed by radiochemotherapy, maintenance therapy with interferon-β (IFNβ) is given for 6 months [9]. IFNβ was integrated into the NPC-GPOH treatment concept after a child with relapsing NPC and multiple intracerebral metastases went into complete remission being treated exclusively with IFNβ [10]. Evidence for a therapeutic benefit of IFNβ in NPC comes also from adults where patients treated with adjuvant IFNβ had a significant better outcome than patients being treated without IFNβ [11]. However, no biological studies have so far been conducted, exploring the effect of IFNβ in NPC cells.

IFNβ belongs as IFNα to the type I interferons which after binding to a common receptor induce through an intracellular signaling cascade the transcription of a number of genes involved in cell proliferation, apoptosis and immune recognition [12]. IFNβ is licensed for the treatment of multiple sclerosis [13]. In contrast, IFNα is used for treatment in various malignancies such as malignant melanoma, chronic myelogenous leukemia and renal cell cancer [14–16]. Its antitumor effect has been shown to rely directly on antiproliferative and proapoptotic effects in tumor cells, but also indirectly on orchestrating an effective anti-tumor immune response [12, 17]. The way of induction of apoptosis by type I interferons in cancer cell lines has been shown to depend on the cellular background. Whereas in neuroblastoma cells, IFNβ induces apoptotic cell death through the intrinsic pathway by downregulation of phosphatidylinositol 3-kinase/AKT signaling, cytochrome C release and activation of procaspase 9 [18], induction of apoptosis in melanoma and breast cancer cells is mediated *via* the extrinsic signaling pathway and dependent on the expression of the death ligand TRAIL [19–20].

In this study we have analyzed the effect of IFNβ on cell death in a panel of NPC cell lines and a nasoepithelial cell line to reveal possible biological mechanisms for its efficacy in the treatment of NPC. Since cell lines in general are genetically unstable, as in the NPC system documented by the loss of EBV during culture [21–22] and therefore might not reflect the biological behavior of originary tumor cells, we have included NPC cells freshly isolated from a patient-derived xenograft in the analyses [23].

## RESULTS

### IFNβ decreases the viability of NPC cells

**Figure 1:**
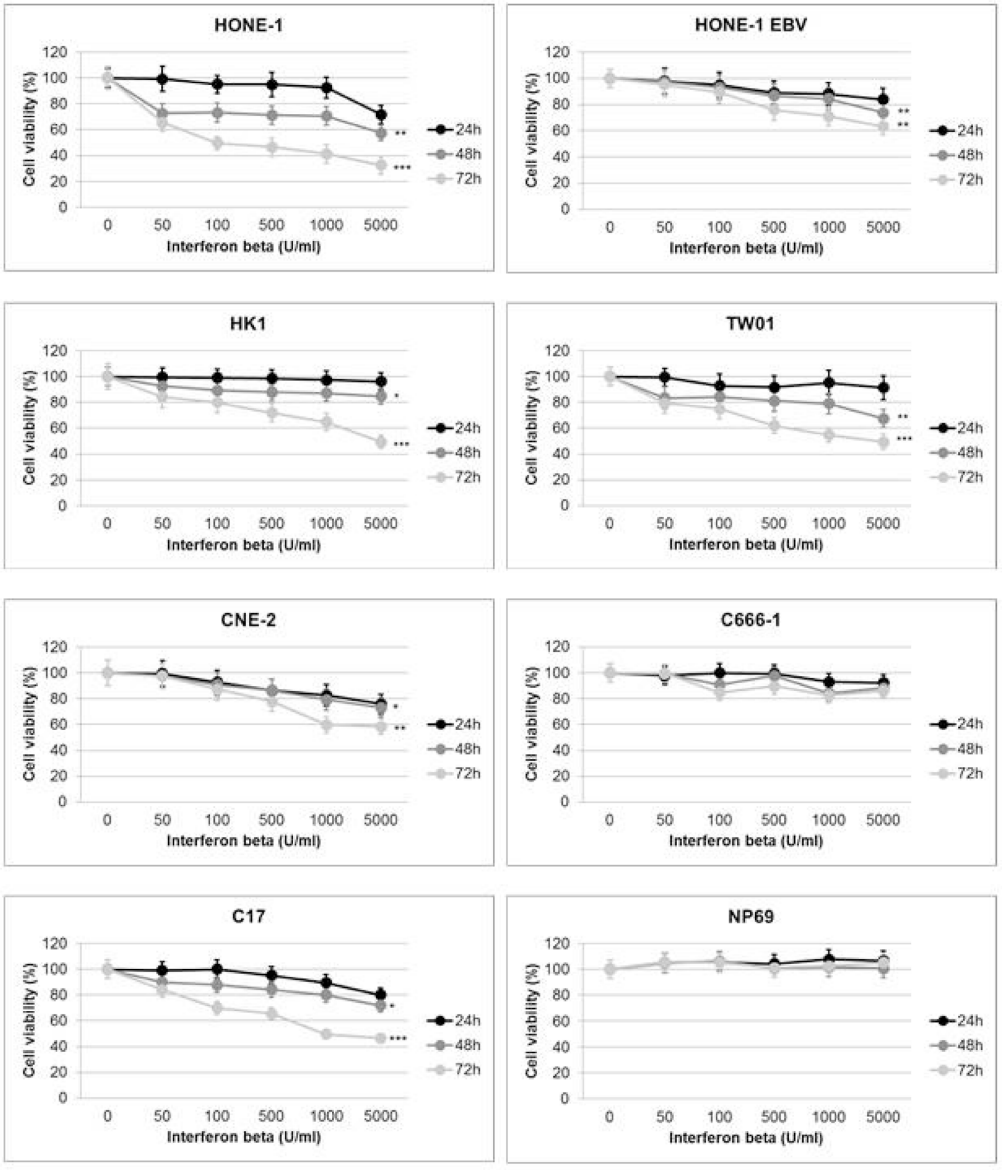
IFNβ decreases viability of NPC cells IFNβ decreases cell viability in a dose-dependent way starting 24 h after incubation. After 48 h and 72 h of incubation with IFNβ a significant reduction in cell viability is observed in NPC cell lines HONE-1, HONE-1 EBV, CNE-2, HK-1, TW01 and C17-PDX cells. No effect is seen in the nasoepithelial cell line NP69 and NPC cell line C666-1. Cell viability was measured by Rotitest Vital. Cells were plated in quintuplicates in 96-well plates. Data are presented as means ± S.E.M., each experiment was done three times (Student's *t*-test; ^*^P < 0.05; ^**^P < 0.01; ^***^P < 0.001).

In a first experiment we investigated the effect of IFNβ on the viability of NPC cells using the WST-8 reduction assay. All cell lines were treated with IFNβ at various concentrations (0–5,000 U/ml) for 24 h, 48 h or 72 h. In humans, serum concentrations of up to 1,000 U/ml can be achieved at therapeutic dosages, e.g. used for the treatment of multiple sclerosis [13, 24]. Incubation with IFNβ for 24 h led to a decrease in cell viability in two NPC cell lines (HONE-1 EBV, CNE-2) starting at a concentration of 500 U/ml and in C17-PDX cells at 1,000 U/ml (Figure 1). When cells were incubated with IFNβ for 48 h, a significant decrease in the number of viable cells was noted in five out of six NPC cell lines and C17-PDX cells, starting at a concentration between 50 and 100 U/ml. The percentage of viable cells further decreased after 72 h incubation with IFNβ, ranging between 40 and 70% at a concentration of 1,000 U/ml in the five sensitive NPC cell lines and C17-PDX cells (HONE-1, HK1, TW01, C17-PDX: *p* < 0.001; HONE-1 EBV, CNE-2: *p* < 0.01). In contrast, no significant decrease in the number of viable cells was seen when cells of the nasopharyngeal epithelial cell line NP69 or NPC cell line C666-1 were treated with IFNβ. Since the decrease in the number of viable cells by IFNβ could be either a consequence of cell death or reduction in cell proliferation, we next performed cell cycle analysis.

### IFNβ induces apoptosis in NPC cells

**Figure 2:**
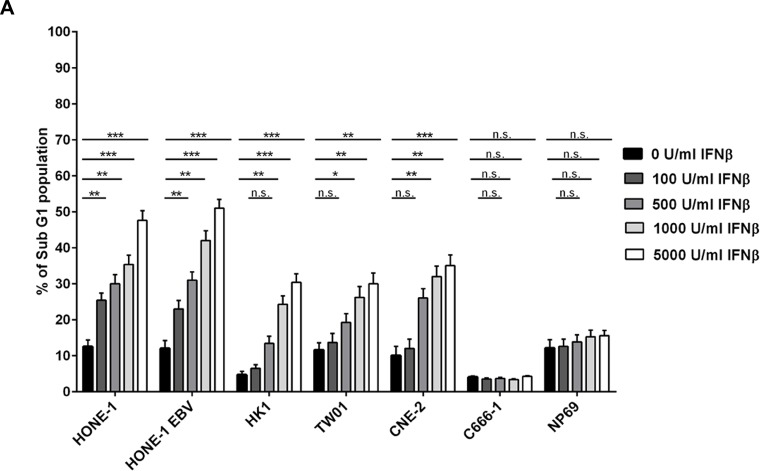

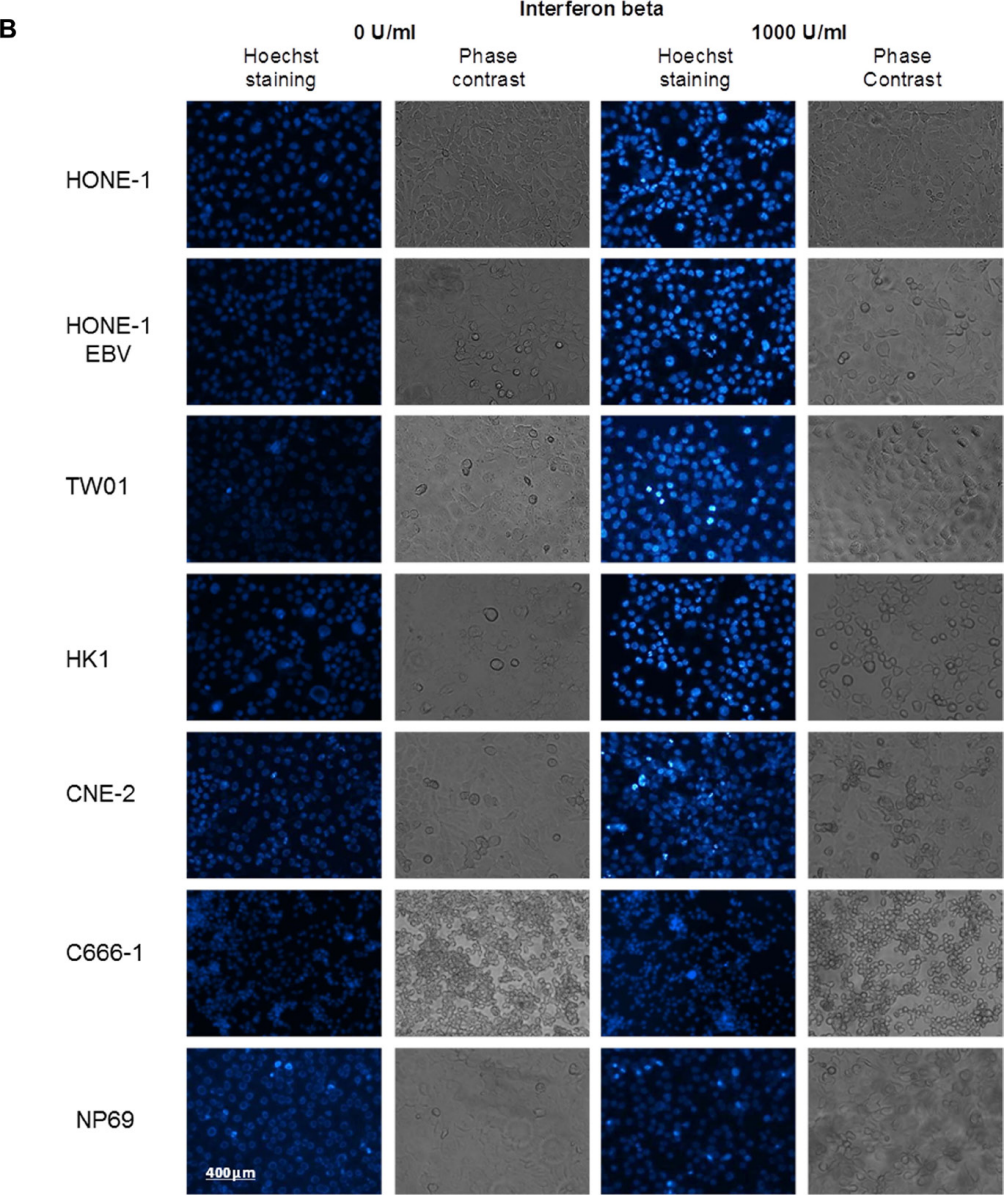

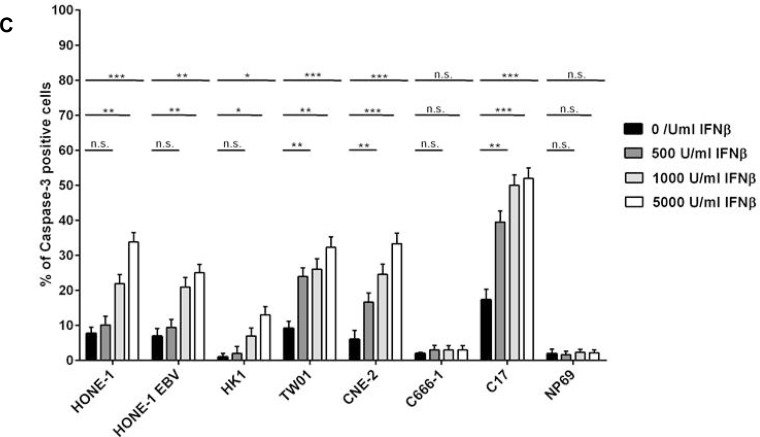
IFNβ induces apoptosis in NPC cells (**A**) Cell cycle analysis. IFNβ induces apoptosis measured by an increase in subG1 in NPC cell lines HONE-1, HONE-1 EBV, CNE-2, HK-1 and TW01. No effect is seen in NPC cell line C666-1 and the nasoepithelial cell line NP69. The data represent the means of three independent experiments and the corresponding standard error. (**B**) Hoechst 33258 staining. Treatment with IFNβ leads to morphological signs of apoptosis (condensed and fragmented nuclei) in NPC cell lines HONE-1, HONE-1 EBV, CNE-2, HK-1 and TW01, but not in cell line C666-1 and the immortalized nasoepithelial cell line NP69. Morphologic changes were examined under a fluorescence microscope at 200× magnification. Phase contrast images are shown to compare for cell density. (**C**) Treatment with IFNβ increases the number of cells with activated caspase-3 in the five IFN-sensitive NPC-cell lines and in C17-PDX cells but not in cell line C666-1 and nasoepithelial cells. Quantitative data are reported as means ± S.E.M. (triplicate samples). Data of all experiments are shown at 72 h after incubation with IFNβ. ^*^p < 0.05; ^**^p < 0.01; ^***^p < 0.001.

NPC cells were treated with different concentration of IFNβ up to 72 h and cell cycle distribution was analyzed by flow cytometry of propidium iodide stained nuclei. Whereas no major effect on cell cycle distribution was noted in any of the cell lines studied, IFNβ induced a significant dose-dependent increase in apoptotic cells in five out of six NPC cell lines (Figure 2A and Supplementary Figure 1). Induction of apoptosis was time- and dose-dependent, starting in cell line CNE-2 at 24 h at a concentration of 5,000 U/ml of IFNβ, and in the other four sensitive cell lines after 48 h of incubation at IFNβ concentrations between 100 and 1,000 U/ml. No induction of apoptosis was observed in the nasoepithelial cell line NP69 and the NPC cell line C666-1.

Apoptosis could also be observed when cells were stained with Hoechst 33258. Here, IFNβ induced morphological changes of apoptosis such as chromatin condensation and nuclear blebbing in the five sensitive NPC cell lines, whereas no such effect was observed in the nasoepithelial cell line NP69 and the NPC cell line C666-1 (Figure 2B).

As the apoptotic phenotype can be induced *via* caspase-dependent but also caspase-independent mechanisms [25], we investigated whether the induction of apoptosis in NPC cells by IFNβ involved the effector caspase-3. Cells were treated as above and stained with an antibody recognizing only the active form of caspase-3. As shown in Figure 2C, IFNβ induced activation of caspase-3 in a dose-dependent way in NPC cell lines HONE-1, HONE-1 EBV, HK-1, TW01, CNE-2 and in C17-PDX cells. No activation of caspase-3 was seen in the nasoepithelial cell line NP69 and NPC cell line C666-1. In summary, these experiments demonstrate that IFNβ induces apoptosis at concentrations achievable in humans in a caspase-dependent way in the majority of NPC cell lines as well as PDX cells but not nasoepithelial cells.

### Induction of apoptosis by IFNβ is dependent on caspase-8

**Figure 3:**
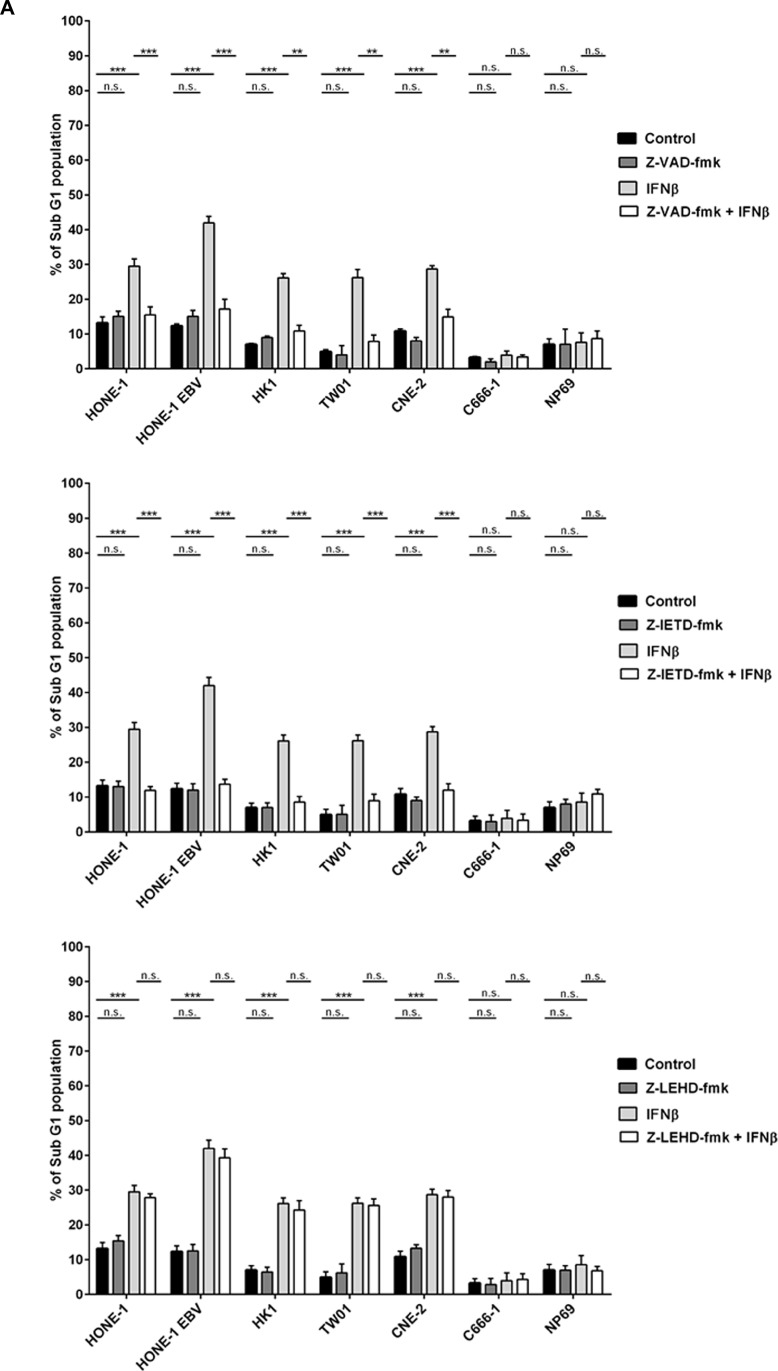

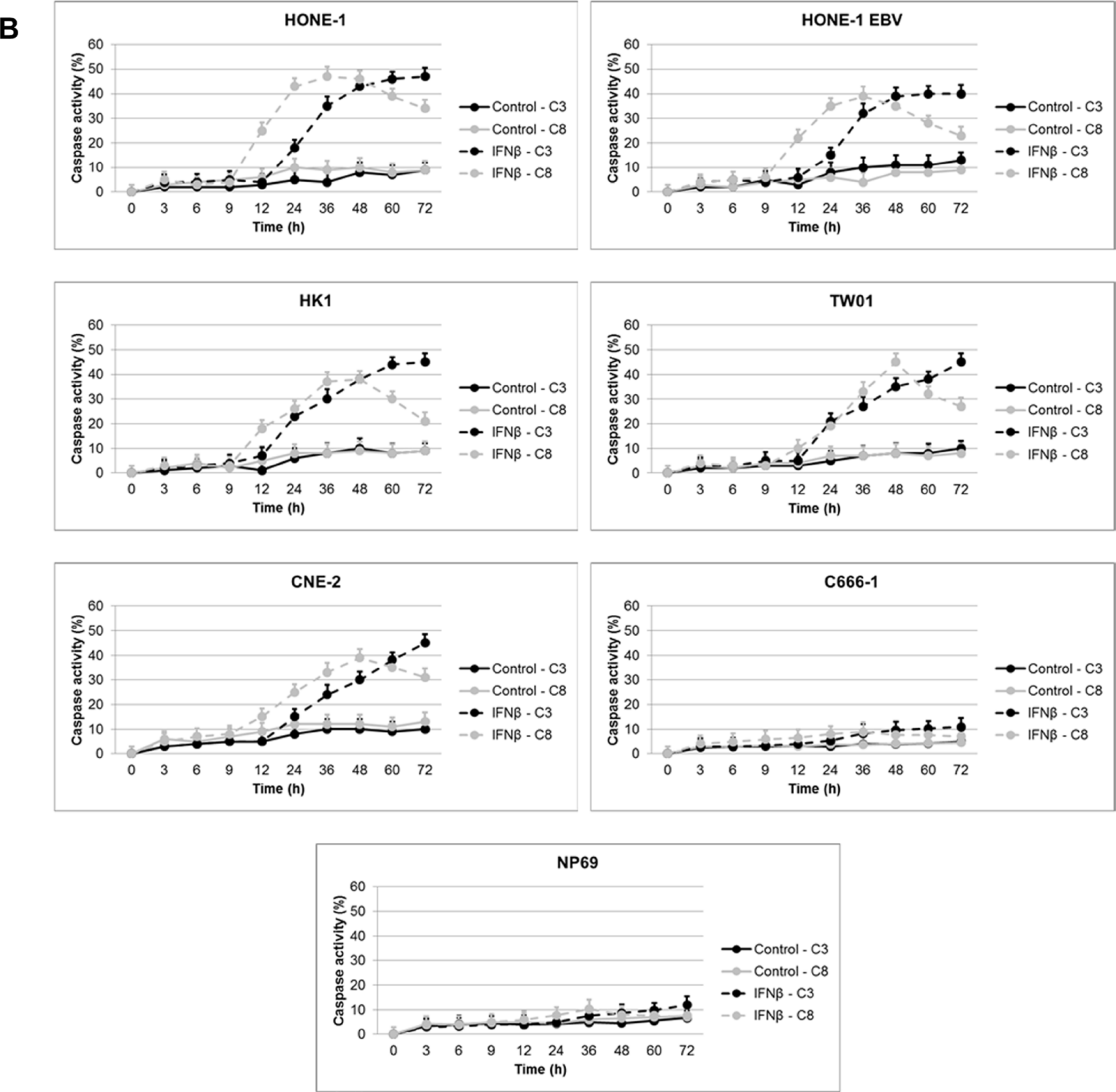

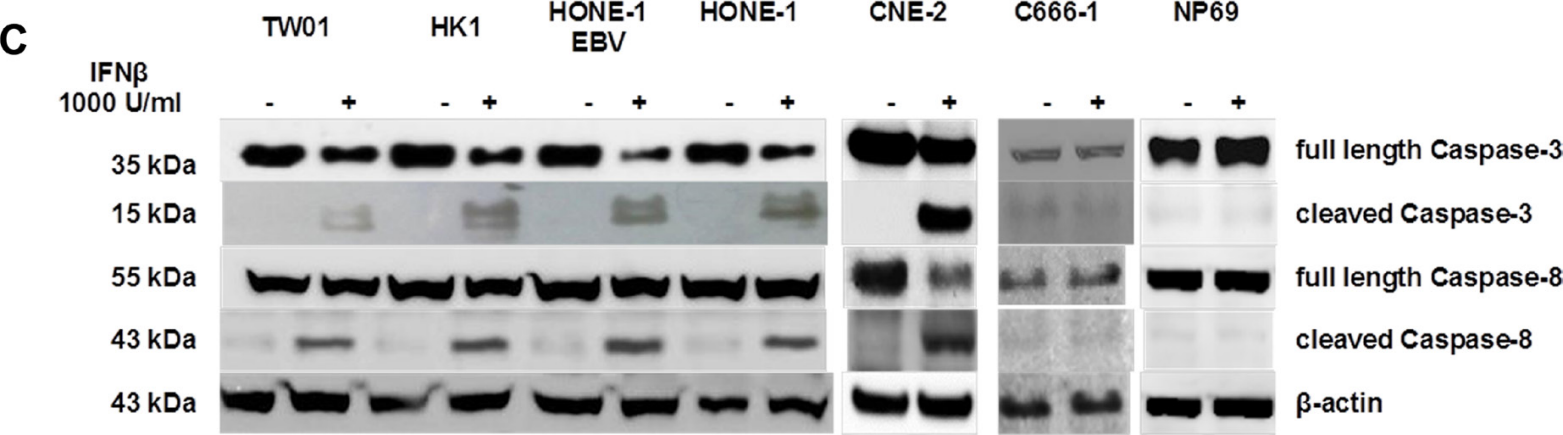
IFNβ induces apoptosis *via* the extrinsic pathway in NPC cells (**A**) Effect of caspase inhibitors on NPC cells treated with IFNβ. Pretreatment of NPC cells with the pan-caspase inhibitor Z-VAD-fmk or the caspase-8 inhibitor Z-IETD-fmk but not the caspase-9 inhibitor Z-LEHD-fmk inhibits IFNβ-mediated apoptosis in IFNβ-sensitive NPC cell lines. Inhibitors were added at 10 μM 1 h prior to IFNβ. Apoptosis was determined by measurement of subG1-content. Data of all experiments are shown at 72 h after IFNβ treatment. Quantitative data are reported as means ± S.E.M. (triplicate samples). ^*^: *p* < 0.05; ^**^: *p* < 0.01; ^***^: *p* < 0.001 (**B**) Activation kinetics of caspases-8, and −3/7 in NPC-cells treated with IFNβ for 72 h. Activation of caspase-8 starts at 12 h of incubation with IFNβ, activation of caspases-3/7 is first noted after 24 h. Caspase activation was measured by the Caspase Glo^®^ assay as described in the method section. Quantitative data are reported as means ± S.E.M. (*n* = 5). (**C**) Immunblot for caspases-8 and −3. Treatment with IFNβ leads to cleavage of caspases-8 and −3 in IFNβ-sensitive NPC cell lines but not in NPC cell line C666-1 and nasoepithelial cell line NP69. Cells were treated for 24 h with 1,000 U/ml IFNβ.

There are two major pathways leading to the activation of effector caspases such as caspase-3, the intrinsic pathway inducing through mitochondrial damage the activation of procapase-9, and the extrinsic pathway in which procaspases-8 or −10 are activated upfront [26]. Using a panel of different caspase-inhibitors we aimed to dissect the effect of different caspases on the induction of apoptosis in NPC cells. Cells of different NPC cell lines and cell line NP69 were treated for 72 h with 1,000 U/ml IFNβ in the presence or absence of the pan-caspase inhibitor Z-VAD-fmk, the caspase-8-inhibitor Z-IETD-fmk or the caspase-9 inhibitor Z-LEHD-fmk. In all IFNβ-sensitive cell lines Z-VAD-fmk and Z-IETD-fmk effectively blocked IFNβ-mediated apoptosis. In comparison, Z-LEHD-fmk did not inhibit IFNβ-mediated apoptosis (Figure 3A). These results, therefore, suggest that IFNβ induces apoptosis in NPC cells *via* activation of the extrinsic but not the intrinsic apoptotic pathway.

As IFNβ induced apoptosis in NPC cells starting at 48 h, we asked at what time point activation of caspases occurred. Activation kinetics of caspase-3/7 and caspase-8 after incubation with IFNβ were studied using a bioluminescent assay. After 12 h of incubation caspase-8 demonstrated significant activation in IFNβ-sensitive cell lines, slightly decreasing after 48 h. Activation of caspase-3 was first observed after 24 h incubation with IFNβ and steadily increased thereafter (Figure 3B). No activation of caspases-3 and −8 was seen in NPC cell line C666-1 and nasoepithelial cell line NP69 indicating that resistance to IFNβ-induced apoptosis in these cells is upstream of the caspase cascade. Activation of caspases 3- and −8 was also demonstrated by detection of cleaved products on immunoblot in IFN-sensitive NPC cell lines but not cell line C666-1 and nasoepithelial cells (Figure 3C).

### The TRAIL signaling pathway is intact in NPC cells

**Figure 4:**
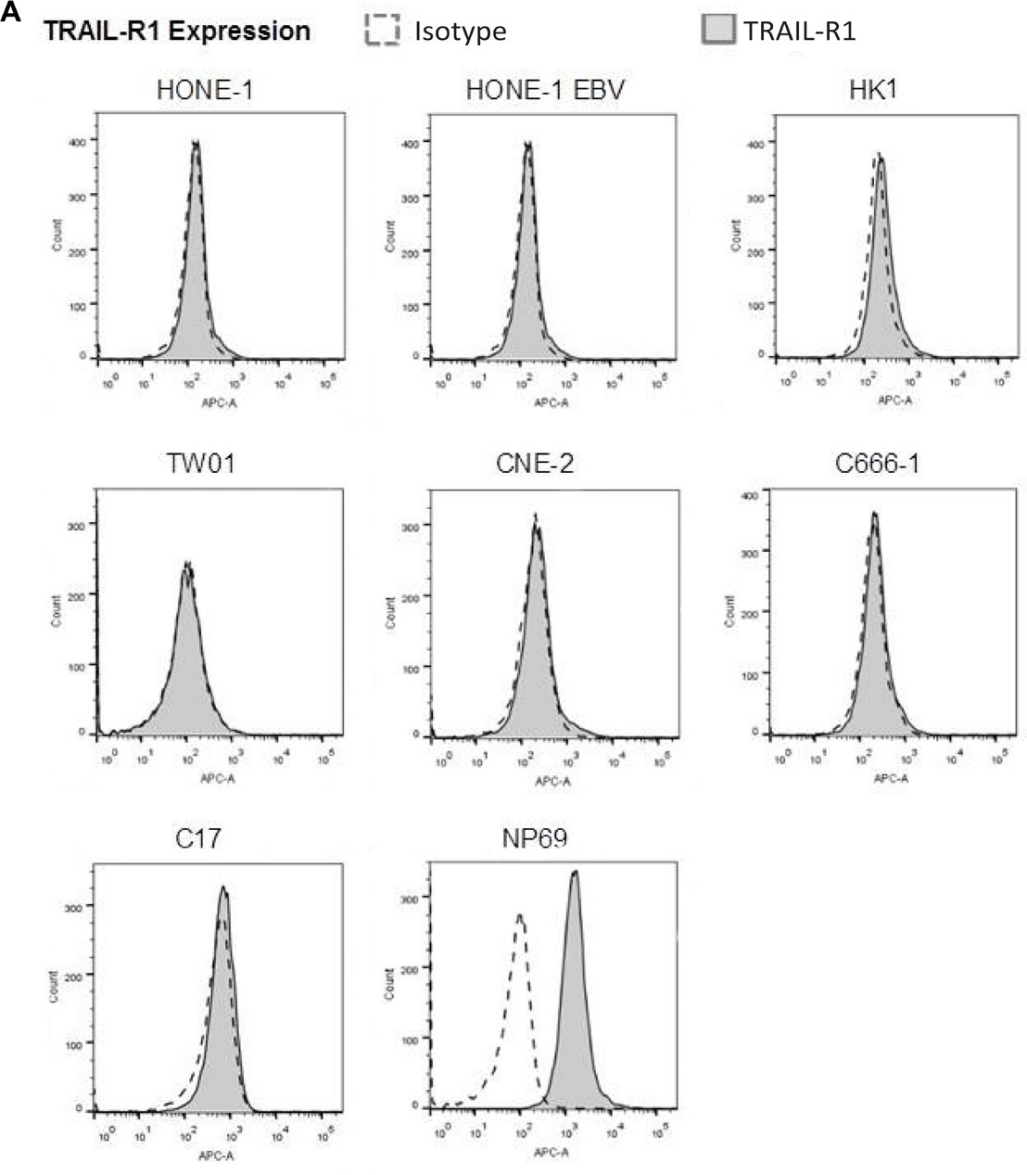

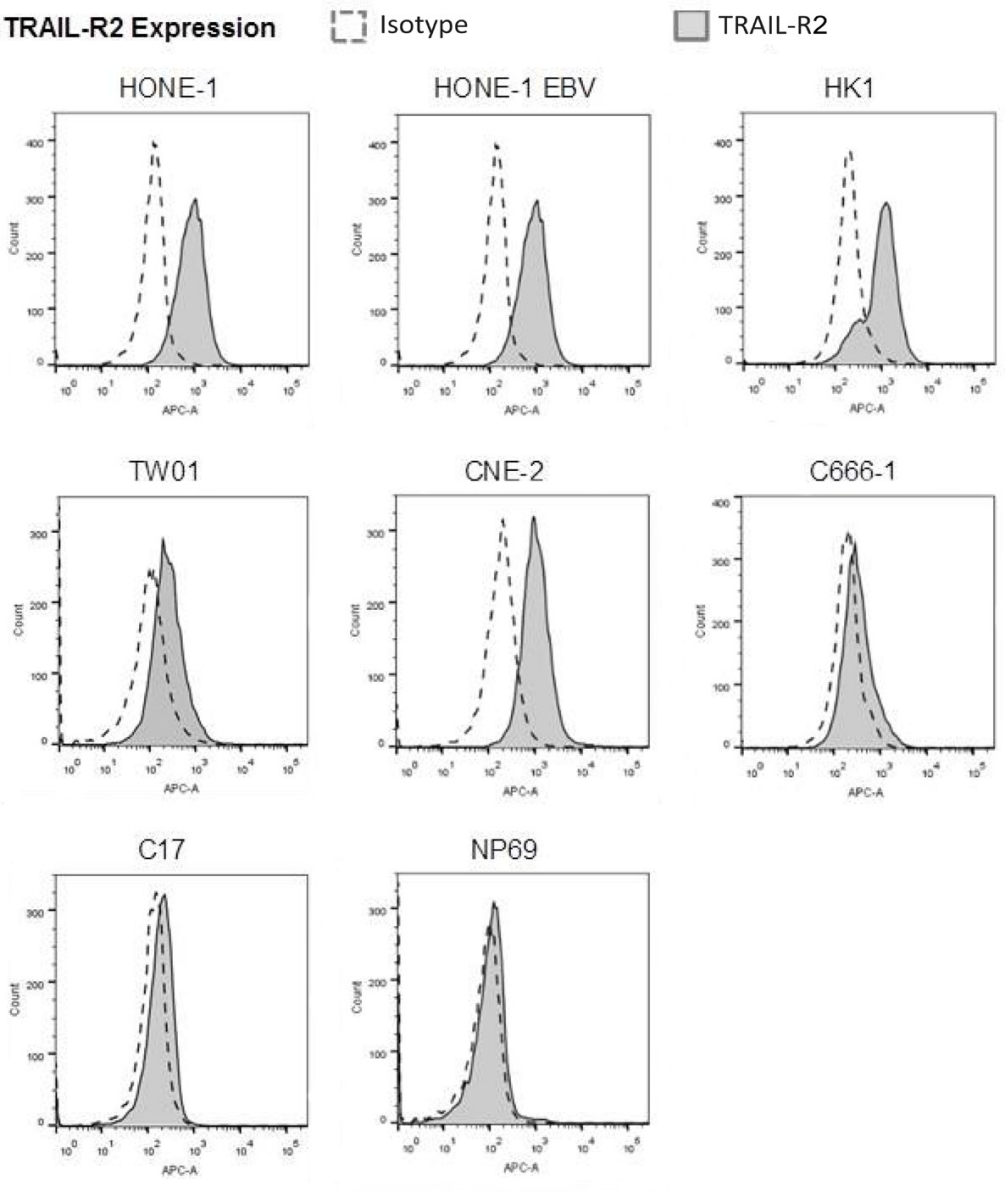

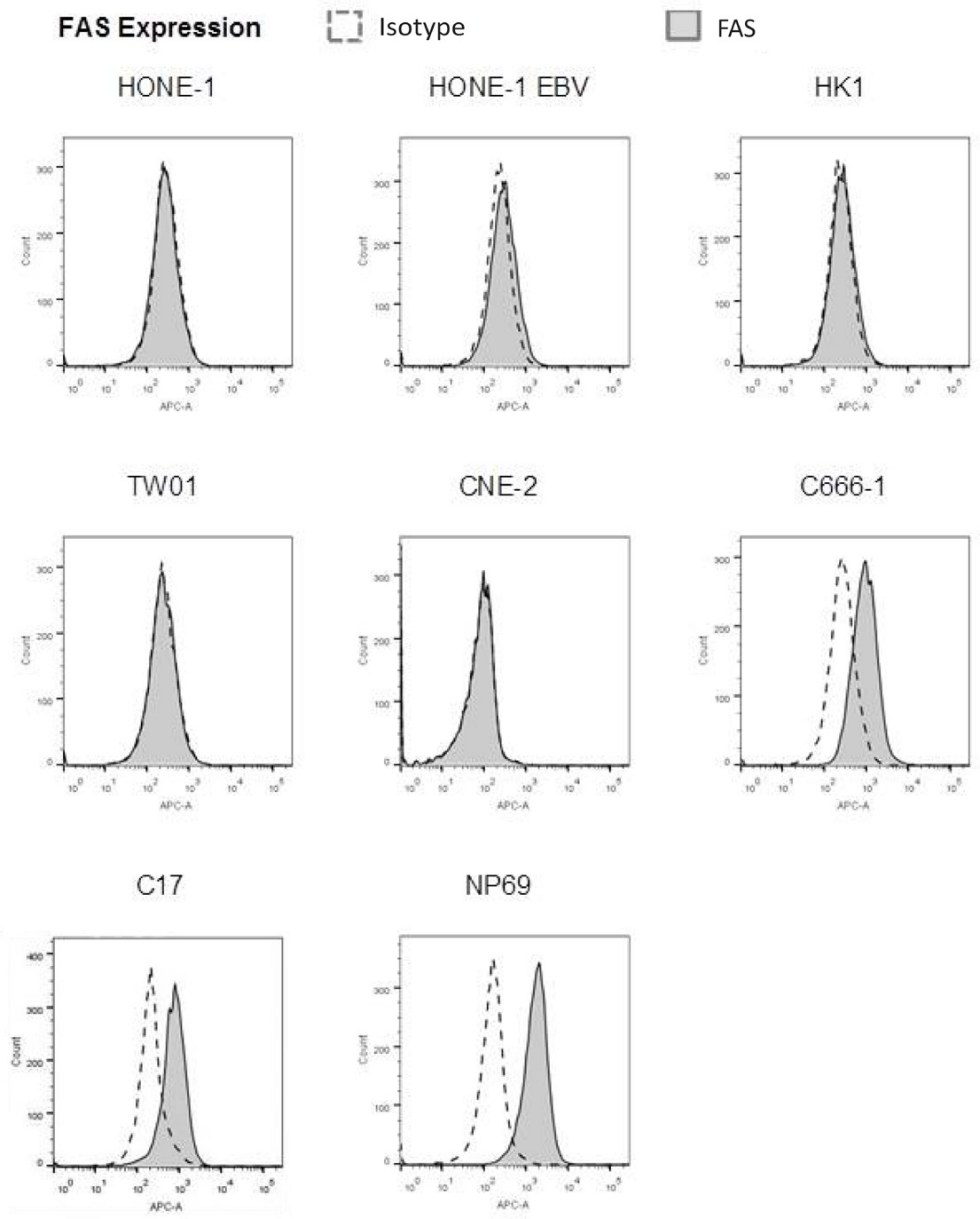

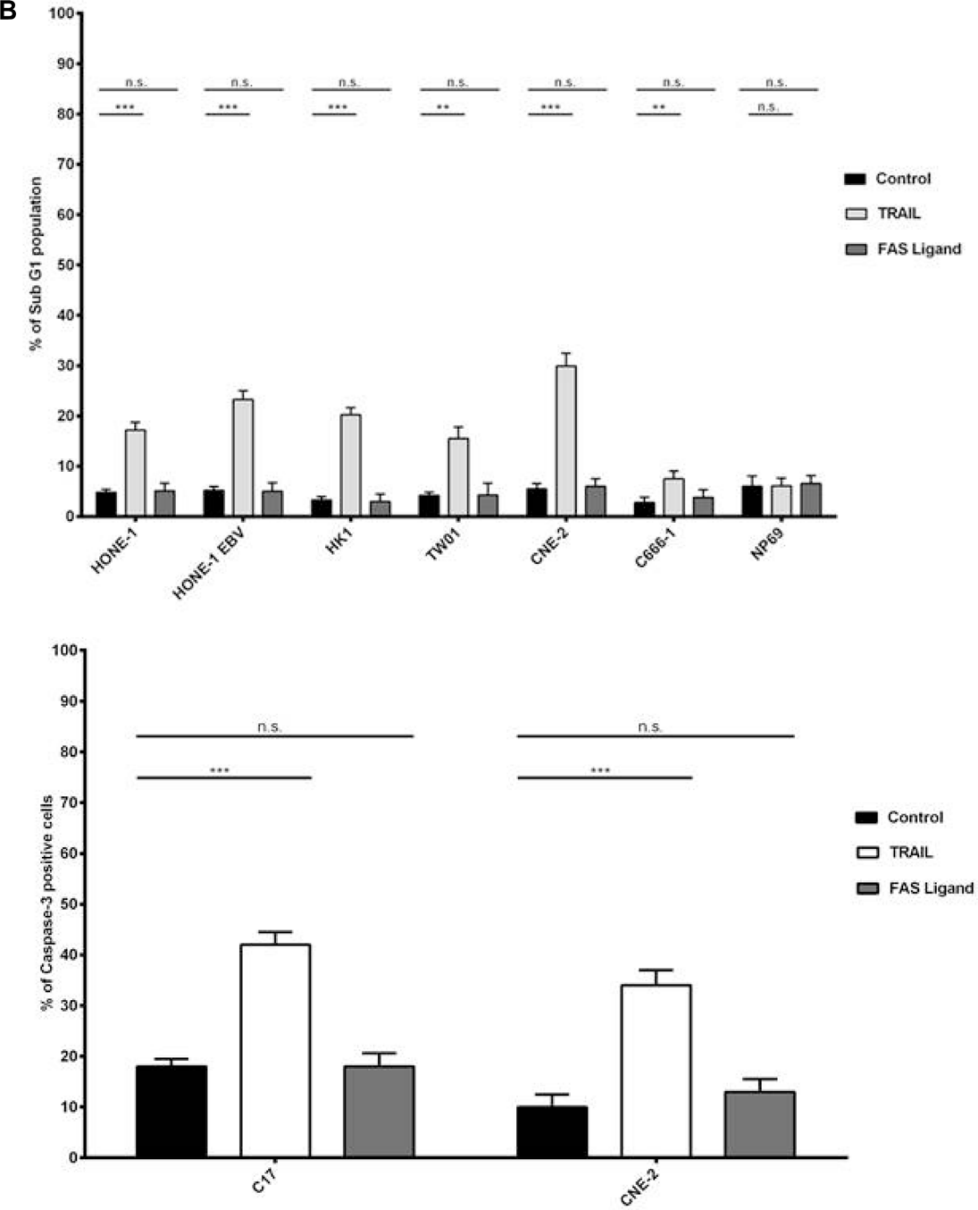

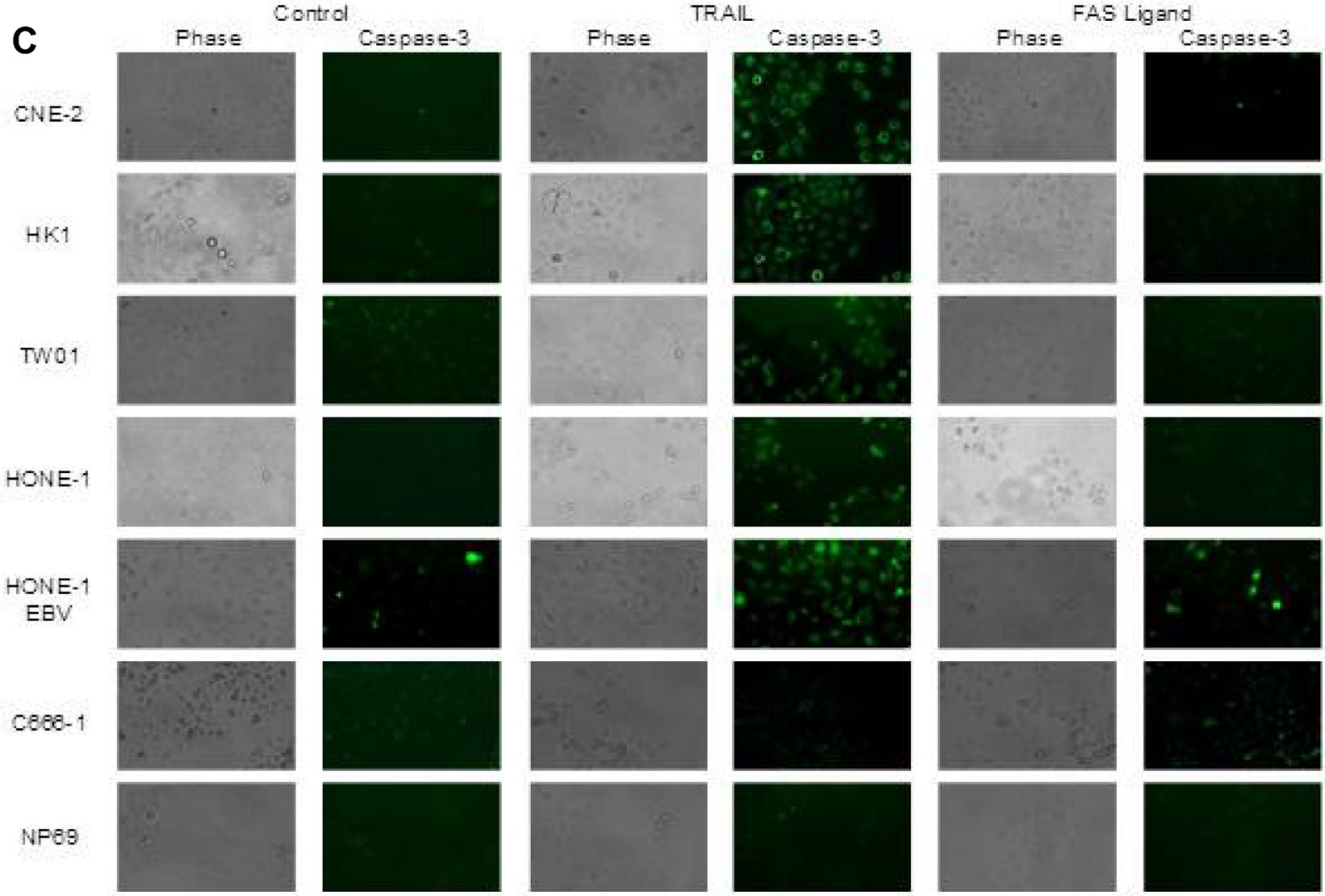

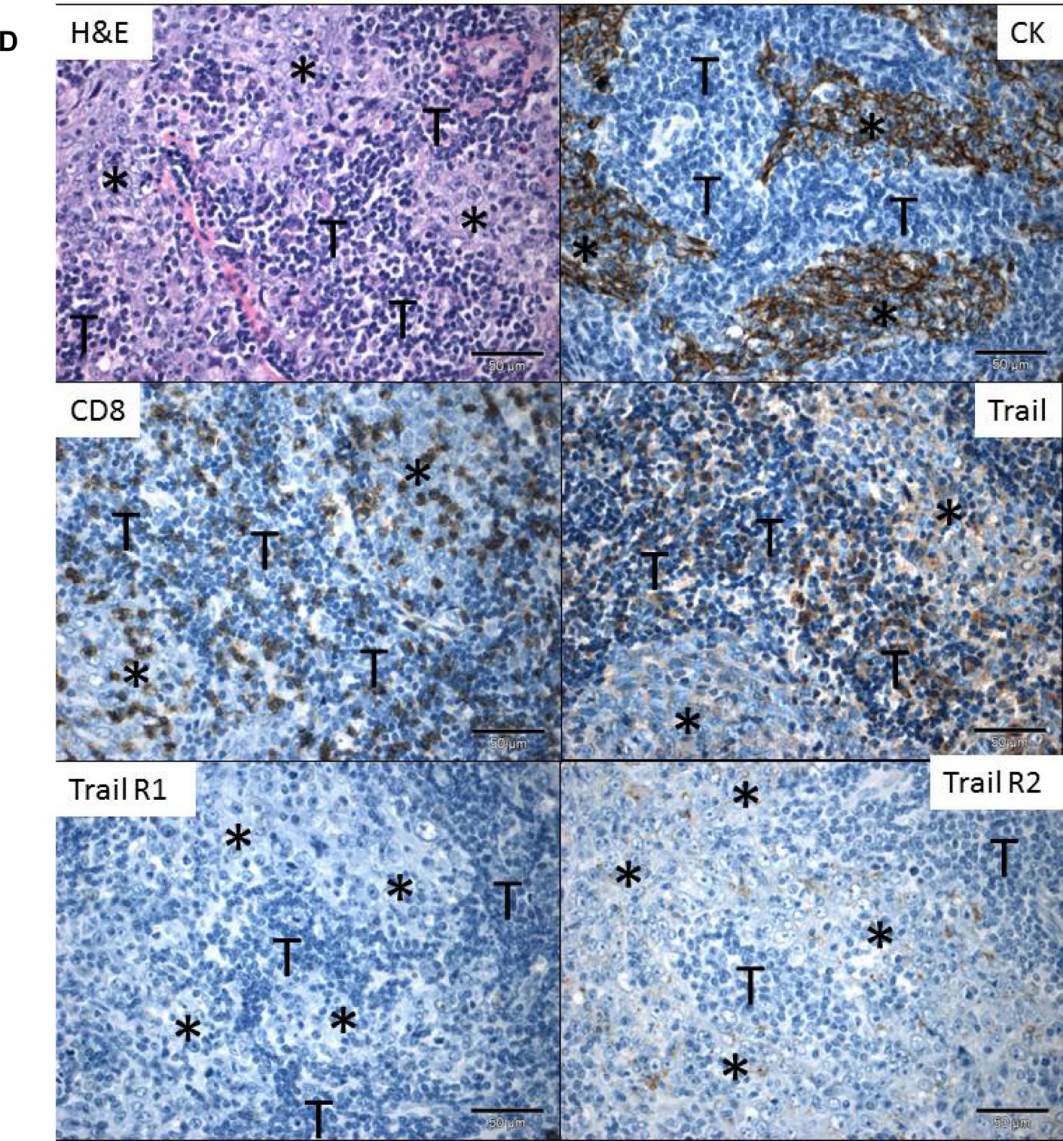

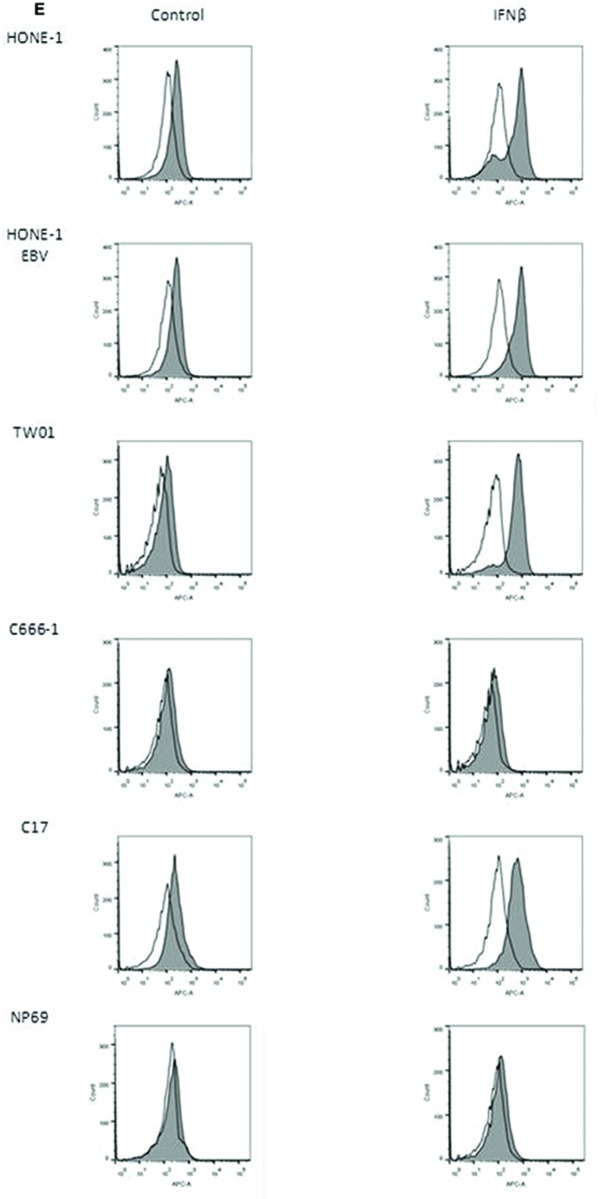
TRAIL induces apoptosis in NPC cells (**A**) Surface expression of TRAIL-R1, TRAIL-R2 and FAS in NPC cells. TRAIL-R2 is expressed in all NPC cell lines but not nasoepithelial cells; low expression in cell line C-666-1 and C17-PDX cells; no expression of TRAIL-R1 in NPC cell lines but nasoepithelial cells. No expression of FAS except in nasopepithelial cells and low in NPC cell line C666-1 and C17-PDX cells. Data were acquired by flow cytometry and were compared to specific isotype controls. (**B**) Induction of apoptosis *via* TRAIL in NPC cell lines but not nasopepithelial cells. Cells were exposed to TRAIL (0.1 μg/ml) or FAS Ligand (0.1 μg/ml) for 24 h. The percentage of apoptotic cells was determined by flow cytometry of cells with subG1-content. Quantitative data are reported as means ± S.E.M. (triplicate samples). ^*^p < 0.05; ^**^p < 0.01; ^***^p < 0.001. (**C**) Immunofluorescence for active caspase-3. Treatment with TRAIL (0.1 μg/ml) for 24 h induced the expression of active caspase-3 in five out of six NPC cell lines. No induction of active caspase-3 was observed when NPC cells were incubated with FAS Ligand. Active caspase-3 was examined under a fluorescence microscope at 200× magnification. (**D**) Immunohistochemistry for TRAIL-R1, TRAIL-R2 and TRAIL in an NPC tumor. NPC cells (^⋆^) stained by cytokeratin, show partially a low to moderate, predominantely membranous, less cytoplasmic expression of TRAIL-R2 and no expression of TRAIL-R1 or TRAIL; a subpopulation of tumor-infiltrating lymphocytes (T), in part stained by CD8, express TRAIL. All images at 400× magnification. Representative images of one NPC tumor are shown; similar staining patterns were obtained in NPC tumors of two other patients analyzed. (**E**) Surface expression of TRAIL-R2 after incubation of cells with IFNβ. Cells were incubated for 72 h with (gray area) or without (white area) 1,000 U/ml IFNβ and then stained and analyzed as in (A). IFNβ upregulated TRAIL-R2 expression in NPC cell lines HONE, HONE-EBV, TW01 as well as C17-PDX cells but not in C666-1 cells and nasoepithelial cells.

Since caspase-8 can be activated after binding of death ligands TRAIL (tumor necrosis factor-related apoptosis inducing ligand) or FASLG (Fas ligand) to their respective receptors TRAIL-R1 and 2 and FAS [27], we were interested whether the TRAIL- and FASLG-signaling pathways were functional in NPC cells. In a first step, we examined the expression of TRAIL-R1 and -R2 and FAS in our panel of NPC cell lines, C17-PDX cells and the nasoepithelial cell line NP69 using flow cytometry. Whereas expression of TRAIL-R1 could not be detected in any of the NPC cell lines studied, we were able to demonstrate high expression of TRAIL-R2 in five out of six NPC cell lines; only weak expression of TRAIL-R2 was observed in cell line C666-1 and C17-PDX cells. Of the six NPC cell lines surface expression of FAS could only be detected at low level in cell line C666-1 and in C17-PDX cells. In contrast, the nasoepithelial cell line did not express TRAIL-R2 but showed surface expression of TRAIL-R1 and FAS (Figure 4A).

We next determined the functionality of the TRAIL/FASLG pathway in NPC cells. Cells were incubated with recombinant human TRAIL (0.1 μg/ml) or FASLG (0.1 μg/ml) for 24 h. Apoptosis was determined *via* flow cytometry of propidium-iodide stained nuclear DNA. Figure 4B demonstrates that all NPC cell lines as well as C17-PDX cells significantly underwent apoptosis after 24 h incubation with TRAIL. Apoptosis involved activation of caspase-3 as shown in Figure 4C. In cell line NP69 no signs of apoptosis were detected, even after 72 h (data not shown). In contrast, all cell lines as well as PDX cells were resistant to induction of apoptosis by FASLG.

We also examined expression of TRAIL-R1, TRAIL-R2 and TRAIL in NPC biopsy specimen from 4 patients by immunohistochemistry. As shown in Figure 4D, similar to the results in the cell line system, in 3/4 patients TRAIL-R2 but not TRAIL-R1 (only in 1/4 patients) was expressed in tumor cells; expression of TRAIL-R2 was of low to moderate intensity and showed a predominant membranous staining pattern. No expression of TRAIL was detected on tumor cells, whereas such expression was found on tumor-infiltrating lymphocytes.

Since in C17-PDX cells expression of Trail-R1 was absent and expression of TRAIL-R2 low, and in a recent analysis on the expression of TRAIL receptors in tumors of 174 patients with NPC, TRAIL-R1 and -R2 were detected only in 29.9% and 36.2% of tumors, respectively [28], we asked whether incubation of NPC cells with IFNβ would increase expression of TRAIL receptors. As shown in Figure 4E and Table 1 IFNβ markedly increased expression of TRAIL-R2 in C17-PDX cells (60% after 72 h) and all NPC cell lines except of cell line C666-1. A weaker induction of expression of TRAIL-R1 was observed in all NPC cell lines including C666-1 and C17-PDX cells. No induction or change of expression of FAS after incubation with IFNβ was noted (data not shown).

**Table 1:** Surface Expression of TRAIL-R1 and -R2 in NPC cells in the presence or absence of IFNβ

	Incubation	0 h		24 h		48 h		72 h	
Cells		Control	IFNβ	Control	IFNβ	Control	IFNβ	Control	IFNβ
HONE-1	TRAIL-R1	0.02 ± 2.1	0.02 ± 1.0	0.04 ± 2.1	0.07 ± 2.8	0.3 ± 0.4	6.1 ± 0.2	5.8 ± 0.01	15.0 ± 0.2
	TRAIL-R2	30.3 ± 3.2	29.1 ± 2.2	29.0 ± 3.1	55.9 ± 3.0	33.0 ± 2.5	72.2 ± 3.3	35.3 ± 4.5	80.3 ± 3.2
HONE-1 EBV	TRAIL-R1	0.03 ± 3.4	0.04 ± 1.9	0.08 ± 2.0	0.05 ± 1.3	0.2 ± 0.3	5.5 ± 0.2	6.6 ± 0.01	13.0 ± 0.5
	TRAIL-R2	28.8 ± 2.9	27.1 ± 1.9	30.7 ± 4.2	48.0 ± 1.9	31.1 ± 1.2	78.0 ± 2.8	29.0 ± 3.2	89.9 ± 2.2
HK1	TRAIL-R1	0.02 ± 2.7	0.03 ± 3.0	0.1 ± 0.1	0.9 ± 0.3	1.1 ± 0.5	12.3 ± 0.4	12.9 ± 0.2	25.0 ± 0.3
	TRAIL-R2	29.9 ± 2.2	32.4 ± 3.0	30.9 ± 2.7	58.0 ± 2.3	35.6 ± 3.1	80.1 ± 3.3	37.7 ± 2.3	88.1 ± 4.1
TW1	TRAIL-R1	0.01 ± 1.3	0.01 ± 2.3	0.1 ± 0.2	0.7 ± 0.2	0.9 ± 0.2	7.0 ± 0.2	7.4 ± 0.2	10.0 ± 0.2
	TRAIL-R2	16.0 ± 2.0	16.3 ± 2.9	16.0 ± 3.2	39.3 ± 5.2	18.0 ± 2.5	69.1 ± 2.1	15.8 ± 1.5	84.4 ± 1.4
CNE-2	TRAIL-R1	0.04 ± 2.2	0.1 ± 3.4	0.1 ± 2.3	0.09 ± 1.8	1.1 ± 0.2	12.0 ± 0.7	13.5 ± 0.1	20.0 ± 0.2
	TRAIL-R2	40.1 ± 3.1	38.4 ± 2.7	47.6 ± 2.3	46.5 ± 3.0	67.2 ± 5.3	68.8 ± 2.3	77.7 ± 3.3	80.8 ± 3.5
C666-1	TRAIL-R1	0.04 ± 3.3	0.05 ± 2.8	0.06 ± 3.8	0.1 ± 3.5	2.0 ± 0.2	5.2 ± 0.1	5.5 ± 0.2	13.0 ± 0.1
	TRAIL-R2	3.0 ± 2.2	4.1 ± 3.4	4.3 ± 4.0	4.5 ± 3.2	3.9 ± 2.2	6.5 ± 2.5	3.9 ± 4.2	6.2 ± 2.5
C17-PDX	TRAIL-R1	0.07 ± 3.4	0.09 ± 2.9	2.2 ± 2.0	10.1 ± 2.2	3.0 ± 0.3	18.1 ± 0.1	13.2 ± 0.2	23 ± 0.5
	TRAIL-R2	6.0 ± 1.9	5.8 ± 2.2	5.9 ± 2.2	37.7 ± 2.7	6.3 ± 2.5	40.2 ± 3.5	5.3 ± 3.3	60 ± 2.5
NP69	TRAIL-R1	29 ± 0.5	32 ± 0.3	28 ± 0.2	28 ± 0.5	31 ± 0.5	31 ± 0.2	29 ± 0.5	30 ± 0.5
	TRAIL-R2	0.05 ± 2.0	0.06 ± 0.9	0.08 ± 1.0	0.04 ± 3.4	4.8 ± 3.5	5.5 ± 3.5	5 ± 2.2	5 ± 2.5

Values are net fluorescent intensities between mean fluorescent intensity of anti-TRAIL-R1 or -R2 antibody and mean fluorescent intensity of respective isotype. Cells were incubated for indicated time periods with or without 1,000 U/ml IFNβ.

### IFNβ induces surface expression of TRAIL in NPC cells

**Figure 5:**
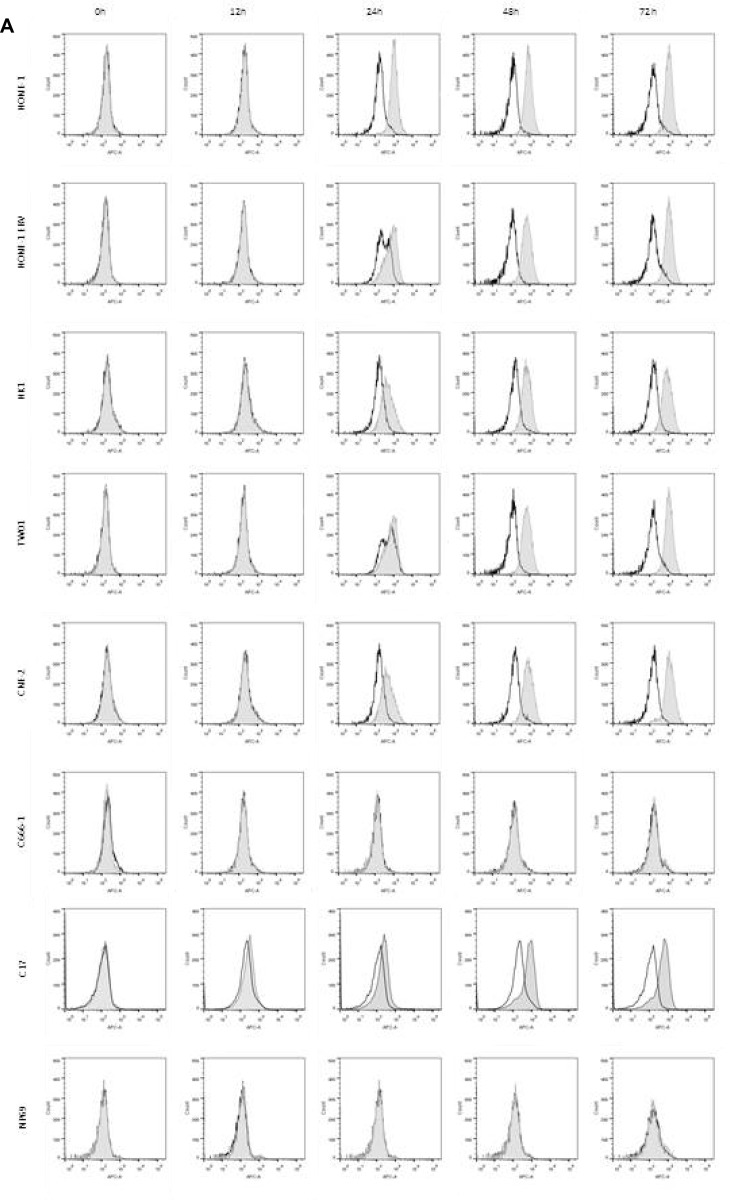

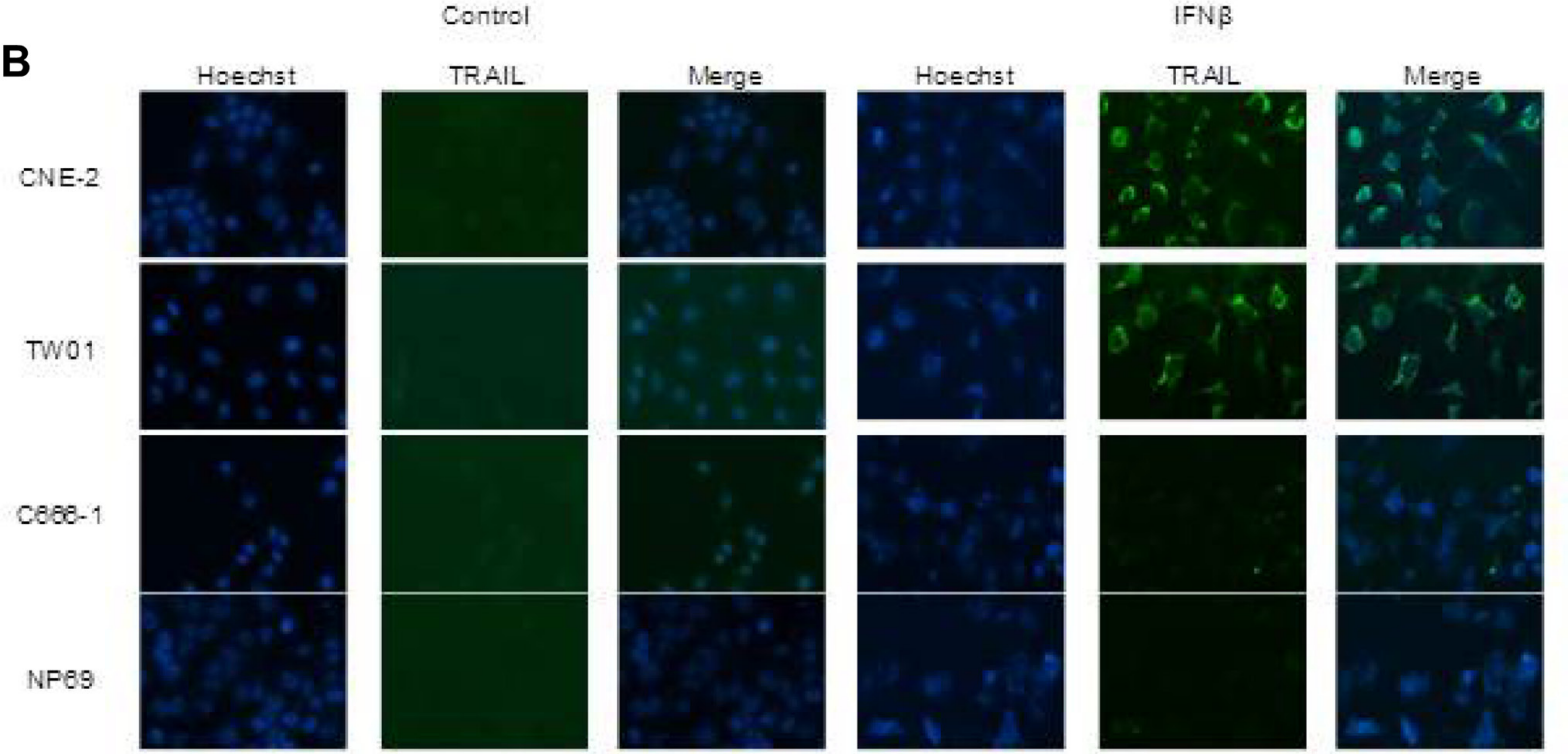
IFNβ induces surface expression of TRAIL in NPC cells (**A**) IFNβ induces surface expression of TRAIL in NPC cell lines HONE-1, HONE-1 EBV, CNE-2, HK-1, TW01 and C17-PDX cells, but not in cell line C666-1 and the immortalized nasoepithelial cell line NP69. TRAIL-expression was first noted 24 h after incubation with IFNβ. Cells were incubated with 1,000 U/ml IFNβ and surface expression was analyzed by flow cytometry comparing IFNβ-treated cells (gray area) against non-treated cells (white area). (**B**) Immunolocalization of TRAIL in NPC cells. Cells were treated for 72 h with IFNβ and stained for TRAIL as described in “Materials and Methods”. Nuclei were counterstained with Hoechst 33258. Confocal microscopy at 400× magnification demonstrates predominant localization of TRAIL on the cell surface.

Since we showed that IFNβ induced apoptosis in NPC cells *via* the extrinsic apoptotic pathway and that the TRAIL-signaling pathway was intact in these cells, we wondered whether IFNβ induced expression of TRAIL in NPC cells. To answer this question, NPC cells were incubated with IFNβ up to 72 h and surface expression of TRAIL was analyzed by flow cytometry (Figure 5A) and by confocal microscopy (Figure 5B). Whereas none of the cell lines expressed TRAIL at baseline, expression of TRAIL was observed starting 24 h after incubation with IFNβ in six of seven NPC cell lines and C17-PDX cells. IFNβ did not induce expression of TRAIL in the nasoepithelial cell line NP69 and NPC cell line C666-1.

### IFNβ induces apoptosis in NPC cells through the TRAIL-signaling pathway

**Figure 6:**
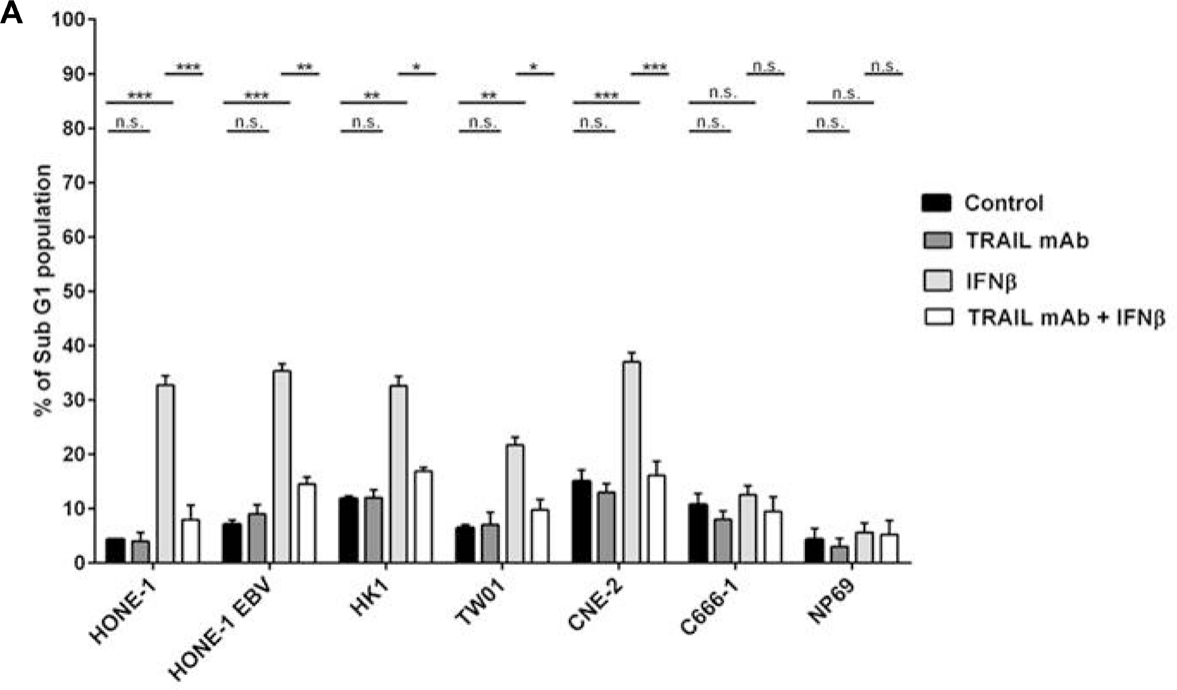

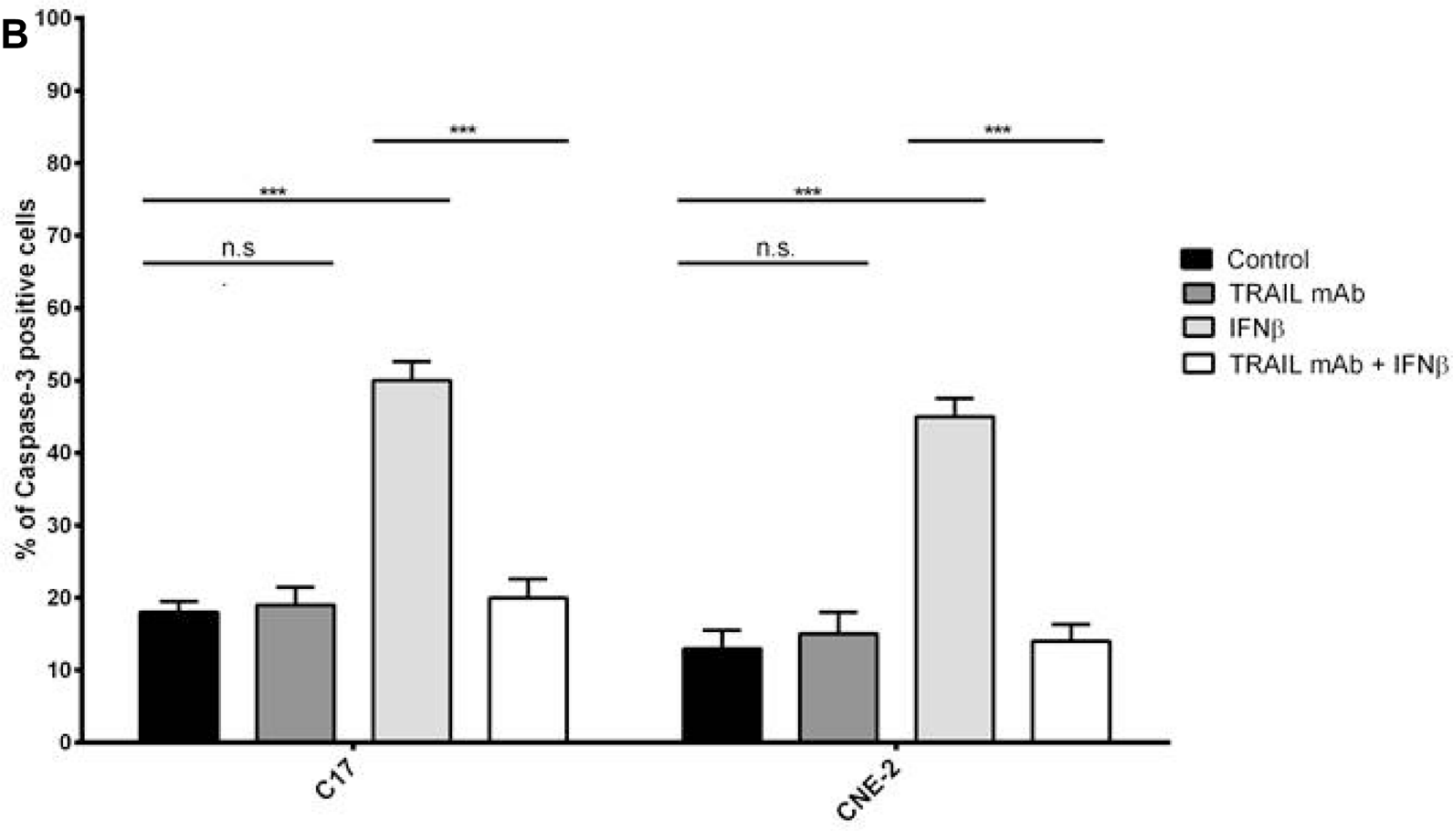

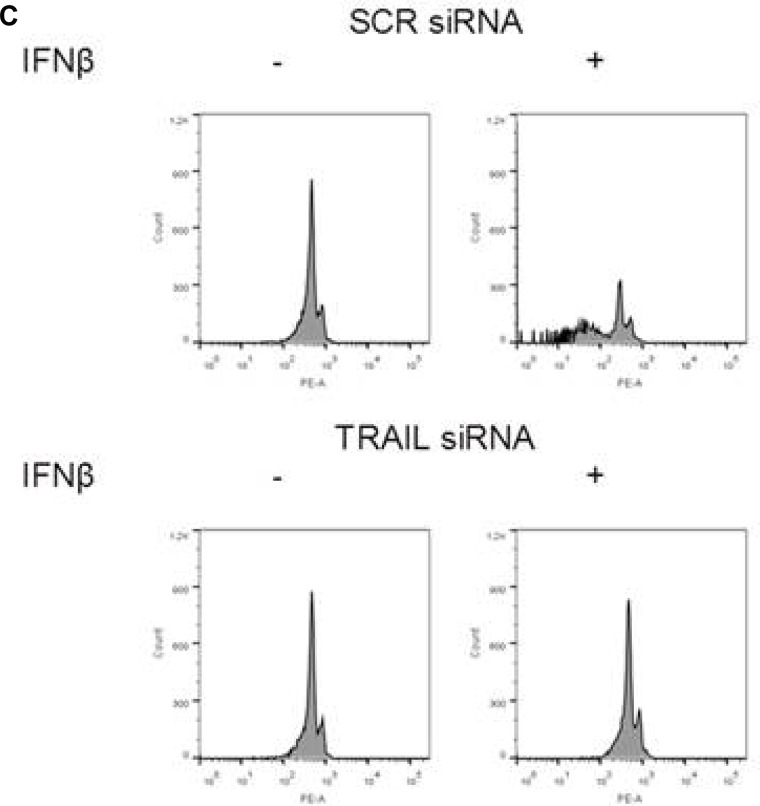

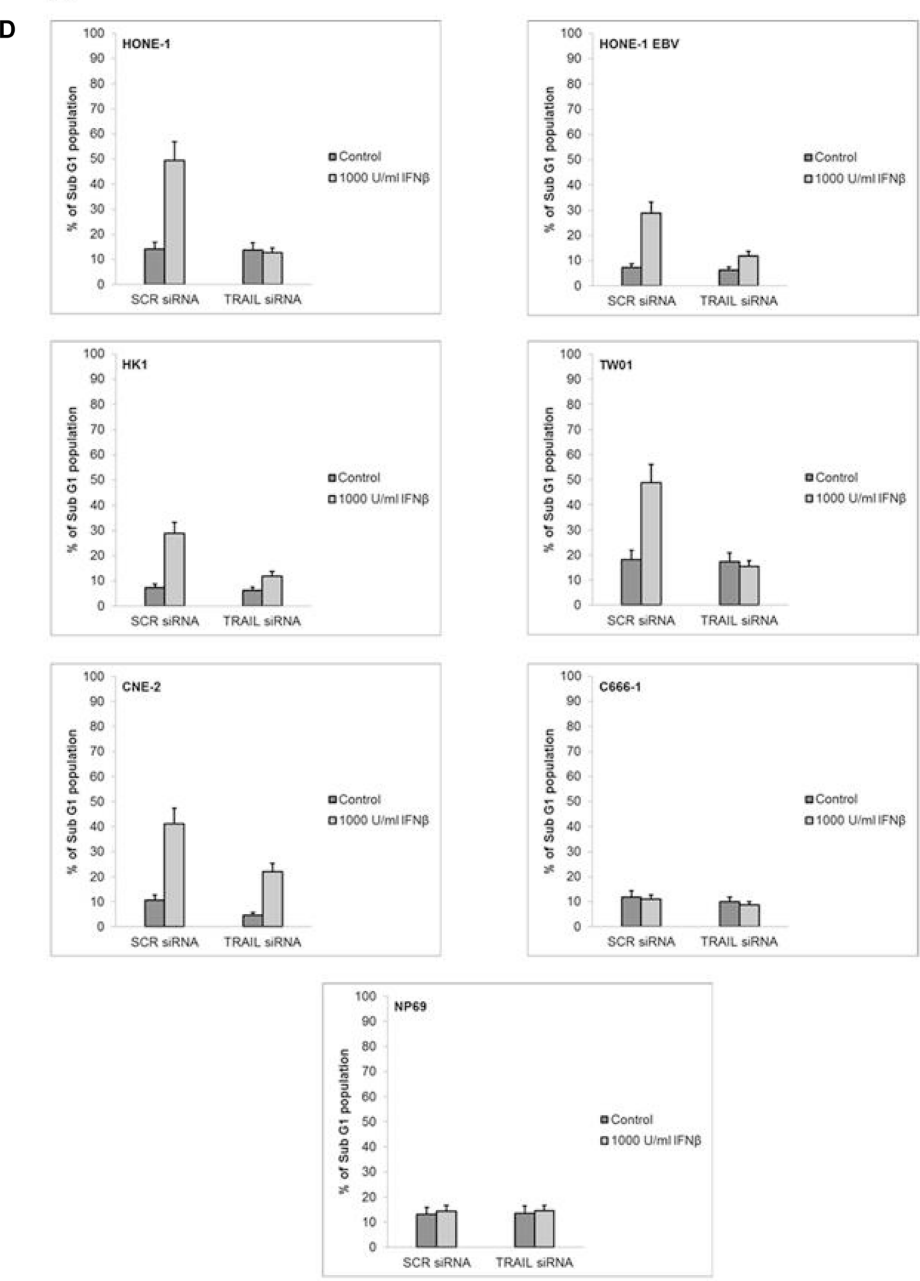
Inhibition of TRAIL inhibits IFNβ-induced apoptosis in NPC cells NPC cells were treated with 1,000 U/ml IFNβ for 72 h in the presence or absence of an anti-TRAIL blocking mAb; apoptosis was then measured by analysis of subG1-content (**A**) or detection of activated caspase-3 (**B**). Anti-TRAIL mAb inhibited IFNβ-induced apoptosis in all IFNβ-sensitive cell lines and C17-PDX cells. Quantitative data are reported as means ± S.E.M. (triplicate samples). (**C**, **D**) Effect of siRNA knockdown of TRAIL on IFNβ-induced apoptosis in NPC cells. Cells were transfected with TRAIL siRNA or scrambled (SCR) siRNA for 16 h and subsequently treated with 1,000 U/ml IFNβ for 72 h. Apoptosis was determined by flow cytometry of subG1-content. TW01 (C). Quantitative data analysis for all cell lines, reported as means ± S.E.M. (triplicate samples) (D).

As IFNβ induced the expression of TRAIL in NPC cells and the TRAIL signaling pathway was intact in these cells, we asked whether endogenous TRAIL was in fact responsible for the induction of apoptosis by IFNβ in NPC cells. Therefore, neutralization experiments were done using an anti-human TRAIL mAb. NPC cells were treated with 1,000 U/ml IFNβ for 72 h in the presence or absence of a neutralizing anti-human TRAIL mAb. Apoptosis was then analyzed measuring the subG1-DNA content of cells by flow cytometry. Whereas IFNβ alone induced apoptosis in all IFNβ-sensitive cell lines and C17-PDX cells, apoptosis was inhibited by coincubation of cells with anti-TRAIL-antibody (Figure 6A and 6B). In order to evaluate whether the induction of apoptosis by IFNβ was based on *de novo* synthesis of TRAIL, TRAIL mRNA-expression was silenced by specific TRAIL-siRNA before treatment of cells with IFNβ. The efficiency of siRNA-knock down was monitored by measuring TRAIL-surface expression after IFNβ treatment *via* flow cytometry (Supplementary Figure 2). As shown in Figure 6C and 6D transfection of NPC cells with TRAIL-siRNA but not scr-RNA abrogated IFNβ-induced apoptosis. These results indicate that IFNβ induces apoptosis in NPC cells *via* the expression of endogenous TRAIL and subsequent activation of the TRAIL-signaling pathway.

## DISCUSSION

In this study we have shown that IFNβ induces apoptosis in the majority of NPC cell lines and in cells of a patient-derived xenograft at concentrations achievable in humans and that the mechanism of induction of apoptosis is based on the expression of TRAIL in NPC cells and subsequent activation of the TRAIL-signaling pathway in an autocrine way.

Interferons are pleiotropic cytokines which can act on tumor cells directly or indirectly *via* immune effector cells [12]. Type I interferons, of which IFNα and β are the most prominent members, act through the type I receptor which is composed of two subunits, IFNAR1 and IFNAR2. Type I interferons have been shown to induce apoptosis in cancer cell lines of various origins in a cell type-specific way. In neuroblastoma cells IFNβ induced apoptotic cell death through the intrinsic pathway by downregulation of phosphatidylinositol 3-kinase/AKT signaling, cytochrome C release and activation of procaspase 9 [18]. In contrast, in melanoma and breast cancer cells IFNβ has been shown to induce apoptosis *via* the extrinsic signaling pathway dependent on the expression of TRAIL [19–20]. In cervical cancer, however, type I interferons induced apoptosis *via* the extrinsic pathway by balancing cFLIP and caspase-8 independent of death ligands [29]. In our experiments, IFNβ-induced apoptosis was caspase-dependent and could be blocked by using inhibitors against caspase-8 but not caspase-9 indicating a role for the extrinsic but not intrinsic apoptotic pathway. As in the melanoma and breast cancer system we could demonstrate that IFNβ upregulated expression of TRAIL and that IFNβ-induced apoptosis could be inhibited by an anti-TRAIL-antibody and TRAIL-siRNA [19–20]. Expression of TRAIL on the surface of NPC cells was noted after 24 h of incubation with IFNβ, activation of caspases-8 and −3 starting at 24 h and 48 h, respectively, and first signs of apoptosis were noted at 48 h. The time kinetic was similar to the one described for melanoma and breast cancer cell lines [19–20]. IFNβ has also been demonstrated to induce TRAIL-surface expression in CD4+ and CD8+ peripheral blood T cells following CD3-stimulation as well as in NK-cells [30–31]. As we have demonstrated that the TRAIL-signaling pathway is intact in the NPC cell lines studied, IFNβ could not only directly by promoting TRAIL-expression on the surface of NPC-cells but also indirectly by expressing TRAIL on immune effector cells, induce apoptosis in NPC cells. This is of importance in cells like NPC cell line C666-1 in which IFNβ does not induce expression of TRAIL and does not directly lead to apoptosis, whereas C666-1 cells are susceptible to apoptosis by exogenous application of TRAIL.

As the biological effects of IFNβ in NPC cells can be obtained at concentrations which are achieved in the serum of patients treated with IFNβ [24, 32], it might very well be that the induction of apoptosis in NPC cells via the TRAIL-signaling pathway could at least contribute to the therapeutic effect of IFNβ observed in patients with NPC. The observation by Wang *et al*. that tumors from a portion of patients with NPC express TRAIL-R1 (29.9%) and TRAIL-R2 (36.6%) and that the expression of TRAIL-R2 was associated with a better survival rate, indicates that the TRAIL-signaling pathway is of importance in the elimination of NPC tumor cells [28]. Our finding that IFNβ markedly increased expression of TRAIL-R2 in NPC cells including PDX cells, hints that IFNβ could strengthen this elimination process *in vivo* making tumor cells susceptible to the induction of apoptosis by TRAIL. That type I interferons induce the expression of TRAIL *in vivo*, has been shown in neutrophils and monocytes of patients with CML treated with IFNα [33]. In addition, in a phase II trial in patients with metastatic melanoma, TRAIL was detectable in the serum of patients after the application of IFNβ indicating that the induction of TRAIL by IFNβ occurred also *in vivo* [34]. Though only one out of seven patients had a clinical regression after IFNβ, TRAIL-serum levels were highest in the responding patient. IFNα is licensed as adjuvant therapy in patients with high-risk melanoma as it extends relapse-free and overall survival [35–36]. In mice, IFNα suppresses the formation of metastases by driving a systemic immune response [37]. In children and adolescents with nasopharyngeal carcinoma, metastatic relapses are the major source of treatment failure [4–7]. As this rate is lowest in the two GPOH-protocols using IFNβ as adjuvant therapy compared to other prospective studies without IFNβ, IFNβ might well have an antimetastatic effect in NPC.

In conclusions, IFNβ induces apoptosis in nasopharyngeal carcinoma cells but not nasoepithelial cells through expression of TRAIL and subsequent activation of the TRAIL signaling pathway. Further studies to analyze the effect of IFNβ on the antitumor activity of immune cells against NPC cells as well as the role of IFNβ on NPC *in vivo* in the mouse xenograft model are warranted.

## MATERIALS AND METHODS

### Cell lines and culture

Six NPC cell lines and one nasopharyngeal epithelial cell line as a control were used in this study. Cell line CNE-2 was kindly supplied by Prof. Pierre Busson (Gustave Roussy Institute, Paris, France) [38]. Cell line HK1 was obtained from Prof. Lo Kwok Wai from the Chinese University of Hong Kong, China [39]. Cell lines HONE-1, EBV positive - HONE-1 (HONE-1 EBV) [40] and the SV40T-antigen immortalized nasopharyngeal epithelial cell line NP69 [41] were provided by Prof. George Tsao (The Chinese University of Hong Kong, Hong Kong, China). Cell line TW01 was supplied by Prof. Chin-Tarng Lin (National Taiwan University Hospital, Taiwan). Cell line C666-1 was obtained from Prof. Fei-Fei Liu, University of Toronto, Canada [42].

Cell lines CNE-2, HK1 and TW01 were cultured in Dulbecco's modified Eagle's Medium (PAN Biotech, Dorset, UK). Cell lines C666-1, HONE-1 and HONE-1 EBV were maintained in RPMI1640 Medium (Gibco, Paisley, UK). Both media were supplemented with 10% fetal bovine serum (Gibco, Paisley, UK), 100 U/ml penicillin and 100 mg/ml streptomycin (Gibco, NY, USA). Cells were cultured in a humidified incubator with 95% air and 5% CO_2_ at 37° C. The nasopharyngeal epithelium cell line NP69 was cultured in keratinocyte-serum free medium (Gibco, NY, USA). All cells were treated with interferon beta (IFNβ), TRAIL, or FAS Ligand as indicated below.

### Patient-derived xenograft

The xenograft C17 was established from a patient with an EBV-positive metastatic NPC by Prof. Pierre Busson, Paris in nude mice [23]. For the experiments described below, single cells suspensions were derived from freshly isolated C17 tumor fragments by collagenase cell dispersion. C17 cells were kept in culture using RPMI1640 Medium (Gibco) supplemented with 25 mM HEPES, 7, 5% fetal bovine serum (Gibco) and 100 U/ml penicillin and 100 mg/ml streptomycin (Gibco).

### Cells authentication: short tandem repeated profiles

In recent years, two studies have shown that various NPC cell lines were contaminated with HeLa cells which are derived from a cervical carcinoma [43–44]. Thus, we investigated the authenticity of NPC cell lines (C666-1, CNE-2, HK-1, HONE-1, HONE-1 EBV, and TW01) and the immortalized nasopharyngeal epithelial cell line (NP69) used in our experiments. DNA of cell lines was extracted and subjected to DNA fingerprinting analysis using the AmpF/STR Identifiler PCR Amplification Kit (Applied Biosystems, Foster City, USA). A total of 15 short tandem repeat (STR) loci (D8S1179, D21S11, D7S820, CSF1P0, D3S1358, TH01, D13S317, D16S539, D2S1338, D19S433, vWA, TPOX, D18S51, D5S818, FGA) and amelogenin were co-amplified in each sample and detected on an ABI Prism 3100 Genetic Analyzer (Applied Biosystems) according to the manufacturer's protocol. Data were analyzed and allele(s) of each locus were determined by GeneScan and Gene-Mapper TM ID Software (Applied Biosystems). Related samples generally yield a result in the 56–100% match range, and unrelated samples in the 0–55% match range [45–46]. STR profiles of NPC cell lines and the nasopharyngeal epithelial cell line NP69 are shown in Supplementary Figure 3. STR profiles for NPC cell lines HONE-1, HONE-1 EBV and CNE-2 showed 66 %, 66% and 65% matching with HeLa cells, respectively, suggesting contamination. In contrast, STR profiles for cell lines HK1, TW01, C666-1 and NP69 matched only between 23% and 47% with HeLa cells, ruling out contamination.

### Reagents

Human recombinant interferon beta (IFNβ) was obtained from R&D System (NY, USA). Soluble, recombinant human TRAIL, Super FAS Ligand, the primary mouse monoclonal antibodies against TRAIL, TRAIL-R1 and TRAIL-R2 were purchased from Enzo Life Science (Paris, France). The caspase inhibitors Z-VAD-fmk, Z-IETD-fmk and Z-LEHD-fmk were obtained from R&D System (Wiesbaden, Germany). Antibodies used for immunoblotting were mouse anti-human β-actin (Cell Signaling, Danvers, MA, USA), mouse anti-human-TRAIL (Enzo Life Science), mouse anti-human caspase-3 (Cell Signaling), and mouse anti-human caspase-8 (Enzo Life Science). The goat anti-mouse IgG secondary antibody was purchased from Santa Cruz Biotechnology (Heidelberg, Germany). The FITC-active caspase-3 apoptosis test was obtained from BD Pharmingen (San Diego, CA, USA). Hoechst 33258 was purchased from Sigma (St. Louis, MO, USA), Rotitest Vital from Roth (Karlsruhe, Germany). Knock-down experiments for TRAIL-RNA were performed using TRAIL-siRNA (Dharmacon, Freiburg, Germany, Cat. Nr. L-011524-00-0010) and scrambled RNA (Dharmacon, Cat. Nr. D-001810-20).

### Cell proliferation assay

The WST-8 reduction assay (Rotitest Vital) was used to determine the effect of IFNβ (0 to 5,000 U/ml treatment for 24, 48 and 72 h) on cell viability. The assay was performed as reported before [47]. Briefly, cells were seeded into 96-well plates at a density of 2,500 cells/well within 200 μl of growth medium. After 24 h of culture, cells were treated with different concentrations of IFNβ and incubated over defined time periods. At the end of the incubation periods 10 μl of WST-8 solution [2-(2-methoxy-4-nitrophenyl)-3-(4-nitrophenyl)-5-(2,4-disulfophenyl)-2H-tetrazolium, monosodium salt] was added into each well. After 4 h the optical density (OD) of each well was measured at 450 nm with a microplate reader (viability = (ODtreat/ODcontrol) × 100%).

### Cell cycle analysis

Propidium-iodide staining of nuclei was used to determine the effect of IFNβ on cell cycle distribution as well as apoptosis by measurement of sub-G1 DNA content. NPC cells (about 70% confluent) were incubated with IFNβ, FAS ligand, TRAIL or medium up to 72 h. Cells were then trypsinized, centrifuged and cell pellets were suspended in 500 μl fluorochrome solution containing 0.1% sodium citrate, 0.1% Triton X-100, 50 mg/l propidium iodide in deionized/distilled water at a cell density of approximately 1 × 10^6^/ml [48]. Samples were incubated about 1 h in the dark at 4° C and then measured by flow cytometry (BD FACS Canto II, San Jose, CA, USA); 10,000 cells per reaction were counted. Results were analyzed by the FlowJo software (FlowJo, Ashland, OR, USA). Three independent experiments were performed for each assay.

### Caspase-3 activity assay

Caspase-3 activity was measured using a caspase-3 assay kit (BD Pharmingen, San Diego, USA) according to the manufacturer's instructions. Cells were treated with IFNβ (0–72 h), washed twice with cold PBS, lysed on ice in 1 ml lysis buffer (Cytofix/Cytoperm™ Fixation and Permeabilization Solution) and further incubated for 20 min on ice. Cell lysates were then centrifuged at 1,000 rpm for 5 min. The pellet was washed twice with Perm/Wash™ Buffer and labeling performed by adding 100 μl of washing buffer containing 20 μl of antibody (BD Pharmingen). The samples were incubated for further 30 min at room temperature, washed in washing buffer and then analyzed by flow cytometry. A negative control sample incubated with FITC-immunoglobulin G was run in parallel. Three independent experiments were performed for each assay.

### Immunofluorescence staining of caspase-3

Cells (5 × 10^4^) were grown on coverslips and treated with TRAIL or FAS ligand for 24 h. Cells then were washed and stained for active caspase-3 protein according to the manufacturer's instructions (Caspase-3 assay kit; BD Pharmingen, San Diego, CA, USA). Fluorescent caspase-3 protein was immediately analyzed by fluorescence microscopy (AMG, Mill Creek, USA).

### Chromatin staining with Hoechst 33258

Induction of apoptosis by IFNβ was also determined by Hoechst staining. Cells were seeded onto 6-well plates in culture medium, incubated at 37° C for 24 h. The cells were then treated in culture medium with varying concentration of IFNβ (0–1,000 U/ml) for 72 h. At the end of the experiment, cells were detached from the plates by trypsinization and collected by centrifugation at 1,000 rpm for 5 min at room temperature. After washing with PBS, cells were stained with 300 μl bis-benzimide (Hoechst 33258; 1 μg/ml in PBS) for 5 min at room temperature. The stained cells were then examined under a fluorescence microscope (AMG, Mill Creek, WA, USA) at 365 nm excitation. The numbers of normal and apoptotic nuclei in randomly selected areas were counted using a 20× objective. Phase contrast images were obtained to compare for cell density.

### Inhibition of apoptosis

To identify the apoptotic pathways induced by IFNβ, NPC cell lines were incubated with different caspase-inhibitors: pan-caspase-inhibitor Z-VAD-fmk (10 μM), caspase-8-inhibitor Z-IETD-fmk (10 μM) and caspase-9-inhibitor Z-LEHD-fmk (10 μM); also, the TRAIL-blocking antibody anti-TRAIL mAb (100 ng/ml) and the FAS-blocking antibody anti-ZB4 mAb (100 ng/ml) were used. Inhibitors were added at the indicated concentrations 1 h prior to the addition of IFNβ (1,000 U/ml). Apoptosis was determined via flow cytometry by the propidium-iodide method [28]. Results were analyzed by the FlowJo software (FlowJo). Three independent experiments were performed for each assay.

### Flow cytometric analysis of death ligand and their receptors

NPC cells untreated or treated as above with IFNβ for the indicated time periods were suspended at a density of 1 × 10^6^ cells in 500 μl of medium and incubated with 5 μl of mouse anti-human TRAIL-R1-, TRAIL-R2-, FAS- and TRAIL-antibody for 1 h on ice. After washing in PBS three times (5 min each), APC-conjugated goat-anti-mouse antibody (1:200) was added to the cell suspensions and incubated for 1 h on ice. Subsequent to rinsing in PBS, samples were analyzed by flow cytometry. Data were analyzed by the FlowJo software (FlowJo). Three independent experiments were performed for each assay.

### Confocal microscopy analysis of TRAIL expression

NPC cells (7.5 × 10^4^) were plated overnight on glass chamber slides (Thermo Fisher Scientific), followed by incubation with IFNβ (1,000 U/ml) for 72 h. Cells were then fixed with 4% paraformaldehyde, incubated with a monoclonal antibody recognizing TRAIL (Alexis Biochemicals, San Diego, CA, USA; 1:200) for 60 min in PBS containing 0.1% Tween 20 and 5 mg/ml BSA (PBST/BSA) followed by 30 min incubation with Alexa Fluor™ 488-conjugated anti-mouse IgG (Invitrogen, Carlsbad, CA, USA; 1:200 in PBST/BSA). Nuclei were stained with Hoechst 33258 as described above. In all cases, imaging was performed with a Zeiss LSM 510 laser scanning confocal/Confocor 2 microscope using a 40x DIC oil immersion objective and LSM 510 software; acquired images were imported into ImageJ (National Institute of Health; http://rsbweb.nih.gov/ij/).

### Immunoblot

Immunoblotting was performed as described before [47]. Briefly, cells were washed and lysed in buffer containing 50 mM Tris-HCl (pH 7.4), 1% NP-40, 0.5% Na-deoxycholate, 0.1% SDS, 150 mM NaCl, 2 mM EDTA, 50 mM NaF and protease and phosphatase inhibitors. Cellular proteins from total cell lysates (20 μg/sample) were separated by sodium dodecyl sulfate–polyacrylamide gel electrophoresis and transferred onto a nitrocellulose membrane. The membranes were probed using immunoblot analyses with mAb to human caspase-3 (1:1,000), caspase-8 (1:500) and Apo2L/TRAIL (1:1,000), followed by incubation with the goat anti-rabbit IgG-antibody for 1 h at room temperature. Equal protein loading was confirmed by reprobing filters with a monoclonal antibody against β-actin. Immunoreactive bands were detected using enhanced chemiluminescence and visualized by autoradiography.

### Caspase-3/7 and 8 activity assay

Caspase-3/7 and caspase-8 activities were determined using the Caspase-Glo 8 and the Caspase-Glo 3/7 assay kits (Promega, Madison, WI, USA) according to manufacturer's instructions. The assay is based on the cleavage of Z-DEVD-amino-luciferin (Caspase Glo 3/7) or Z-LETD-amino-luciferin (Caspase-Glo 8) by activated caspases-3/7 or −8, respectively, and subsequent generation of a luminescent signal by luciferase. Briefly, NPC cells were seeded at a density of 1.5 × 10^4^ cells per well in a 96-well assay plate overnight. IFNβ was then added for 3, 6, 9, 12, 24, 48 and 72 h. At the end of the experiment, the respective Caspase-Glo reagent was added to each well at a ratio of 1:1 with cell culture media and samples were incubated with gentle agitation for 1 h at room temperature, protected from light. Luminescence of samples was then measured by a luminescence reader (TECAN Infinite 200 Pro, Tecan, Männedorf, Switzerland). Caspase activation was normalized to positive samples treated with recombinant caspase enzyme by dividing the raw luminescence units (RLUs) of IFNβ samples by the RLU value of positive controls × 100%.

### Immunohistochemistry

For immunohistochemical staining, 3 μm sections of formalin fixed, paraffin-embedded tissue samples were deparaffinized by xylene and rehydrated by decreasing concentrations of ethanol. After heat-induced antigen retrieval by pH6 EDTA buffer (DAKO, Carpinteria, CA, USA), endogenous peroxidase activity was deactivated by 3% hydrogen peroxide. Nonspecific protein binding sites were blocked by Protein Block (DAKO, Carpinteria, CA, USA). Mouse anti-human TRAIL-R1 monoclonal antibody (Enzo Life Science, Clone TR1.02), mouse anti-human TRAIL-R2 monoclonal antibody (Enzo Life Science, Clone DJR2-2) and mouse anti-human TRAIL monoclonal antibody (R&D System, Clone 75402) as well as mouse anti-pan-Keratin and anti-CD8 antibody (DAKO) were incubated with the slides for 60 min. For detection, the polymere-based Envision Kit by DAKO (Carpinteria, CA, USA) was applied, including a secondary antibody and DAB (diaminobenzidine) for staining. After counterstain by hematoxylin, dehydration and coverslipping, stained sections were evaluated and histological photographs were taken using a Zeiss microscope (Zeiss, Oberkochen, Germany) and Olympus camera and analysis software (Olympus, Hamburg, Germany).

### Transfection of siRNA

NPC cells were seeded at 10^5^ cells/well in 24-well plates. The following day, when cells reached about 80% confluency, culture medium was aspirated, cells washed with PBS, followed by transfection with Lipofectamine (Invitrogen, Carlsbad, CA, USA) of siRNA against TRAIL or scrambled siRNA. After 16 h the transfection mix was replaced with normal growth medium and the cells were treated with 1,000 U/ml IFNβ for indicated time periods. Transfection efficiency was monitored with measuring surface expression of TRAIL by flow cytometry. Three independent experiments were performed for each assay.

### Statistical analysis

Experimental results were reported as a mean of at least three independent experiments conducted in quintuplicates for cell viability assays and triplicates for flow cytometric analyses. Data in bar graphs were represented as mean ± S.E. Student's *t*-test was used to compared two sets of data with *p* < 0.05 as statistically significant.

## SUPPLEMENTARY MATERIALS FIGURES AND TABLES



## Abbreviations

NPC: nasopharyngeal carcinoma
EBV: Epstein-Barr virus
IFNβ: interferon beta
TRAIL: Tumor Necrosis Factor-Related Apoptosis Inducing Ligand
FASLG: Fas ligand
PDX: patient-derived xenograft.
