# Correction: Interferon beta induces apoptosis in nasopharyngeal carcinoma cells via the TRAIL-signaling pathway

**DOI:** 10.18632/oncotarget.28464

**Published:** 2023-08-30

**Authors:** Anna Makowska, Lora Wahab, Till Braunschweig, Nikiforos-Ioannis Kapetanakis, Christian Vokuhl, Bernd Denecke, Lian Shen, Pierre Busson, Udo Kontny

**Affiliations:** ^1^Division of Pediatric Hematology, Oncology and Stem Cell Transplantation, Medical Faculty, RWTH Aachen University, Aachen, Germany; ^2^Institute of Pathology, Medical Faculty, RWTH Aachen University, Aachen, Germany; ^3^CNRS UMR 8126, Gustave Roussy and Université Paris-Sud/Paris-Saclay, Villejuif, France; ^4^Institute of Pathology, Kiel Pediatric Tumor Registry, Christian-Albrechts-University, Kiel, Germany; ^5^IZKF, Medical Faculty, RWTH Aachen University, Aachen, Germany

**This article has been corrected:** During the assembly of Figure 5B, the file of the immunofluorescence experiment in Figure 3B of another paper [1] was opened for comparison of quality, and this image was mistakenly inserted. In Figure 5B, in each row, the three images to the left as well as the three images to the right are taken from an identical camera position respectively, with different fluorescence filters (1. blue: Hoechst, 2. green: TRAIL, 3. Merge); therefore, all 3 images to the right of cell line C666-1 show the same cells. The corrected Figure 5B, using images from the original data, is shown below. In addition, the images in Figure 5B are mistakenly described as being made by a confocal microscope. However, they were made by an immunofluorescence microscope. Accordingly, the term “confocal microscopy” has been corrected using “immunofluorescence microscopy” in the Method section, Results section and Figure Legend 5. The correct microscope (AMG Evos fl) has now been cited in the Method section as well. In Figure 4A, the flow cytometry images for cell lines HONE-1-EBV TRAIL receptors 1 and 2 have been accidentally duplicated during assembly of the figure from the ones of cell line HONE-1 in the same figure. The corrected Figure 4A, obtained using the original data, is shown below. In Figure 4E, the flow cytometry image for cell line NP69 IFNß has been accidentally duplicated during assembly of the figure from the flow cytometry image for cell line C666-1 Control in the same figure. The corrected Figure 4E, obtained using the original data, is also shown below. The authors declare that these corrections do not change the results or conclusions of this paper.

## MATERIALS AND METHODS

### Immunofluorescence microscopy analysis of TRAIL expression

NPC cells (7.5 × 10^4^) were plated overnight on glass chamber slides (Thermo Fisher Scientific), followed by incubation with IFNβ (1,000 U/ml) for 72 h. Cells were then fixed with 4% paraformaldehyde, incubated with a monoclonal antibody recognizing TRAIL (Alexis Biochemicals, San Diego, CA, USA; 1:200) for 60 min in PBS containing 0.1% Tween 20 and 5 mg/ml BSA (PBST/ BSA) followed by 30 min incubation with Alexa Fluor^™^ 488-conjugated anti-mouse IgG (Invitrogen, Carlsbad, CA, USA; 1:200 in PBST/BSA). Nuclei were stained with Hoechst 33258 as described above. In all cases, imaging was performed with a AMG Evos fl microscope using a 40× DIC oil immersion objective; acquired images were imported into ImageJ (National Institute of Health; http://rsbweb.nih.gov/ij/).

## RESULTS

### IFNβ induces surface expression of TRAIL in NPC cells

Since we showed that IFNβ induced apoptosis in NPC cells via the extrinsic apoptotic pathway and that the TRAIL-signaling pathway was intact in these cells, we wondered whether IFNβ induced expression of TRAIL in NPC cells. To answer this question, NPC cells were incubated with IFNβ up to 72 h and surface expression of TRAIL was analyzed by flow cytometry (Figure 5A) and by immunofluorescence microscopy (Figure 5B). Whereas none of the cell lines expressed TRAIL at baseline, expression of TRAIL was observed starting 24 h after incubation with IFNβ in six of seven NPC cell lines and C17-PDX cells. IFNβ did not induce expression of TRAIL in the nasoepithelial cell line NP69 and NPC cell line C666-1.

Original article: Oncotarget. 2018; 9:14228–14250. 14228-14250. https://doi.org/10.18632/oncotarget.24479

**Figure 4:**
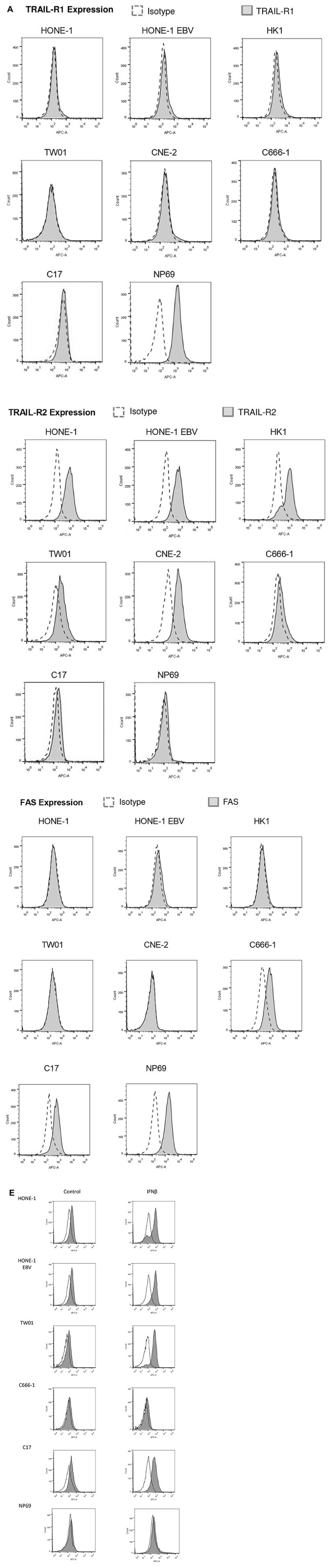
TRAIL induces apoptosis in NPC cells. (**A**) Surface expression of TRAIL-R1, TRAIL-R2 and FAS in NPC cells. TRAIL-R2 is expressed in all NPC cell lines but not nasoepithelial cells; low expression in cell line C-666-1 and C17-PDX cells; no expression of TRAIL-R1 in NPC cell lines but nasoepithelial cells. No expression of FAS except in nasopepithelial cells and low in NPC cell line C666-1 and C17-PDX cells. Data were acquired by flow cytometry and were compared to specific isotype controls. (**E**) Surface expression of TRAIL-R2 after incubation of cells with IFNβ. Cells were incubated for 72 h with (gray area) or without (white area) 1,000 U/ml IFNβ and then stained and analyzed as in (A). IFNβ upregulated TRAIL-R2 expression in NPC cell lines HONE, HONE-EBV, TW01 as well as C17-PDX cells but not in C666-1 cells and nasoepithelial cells.

**Figure 5:**
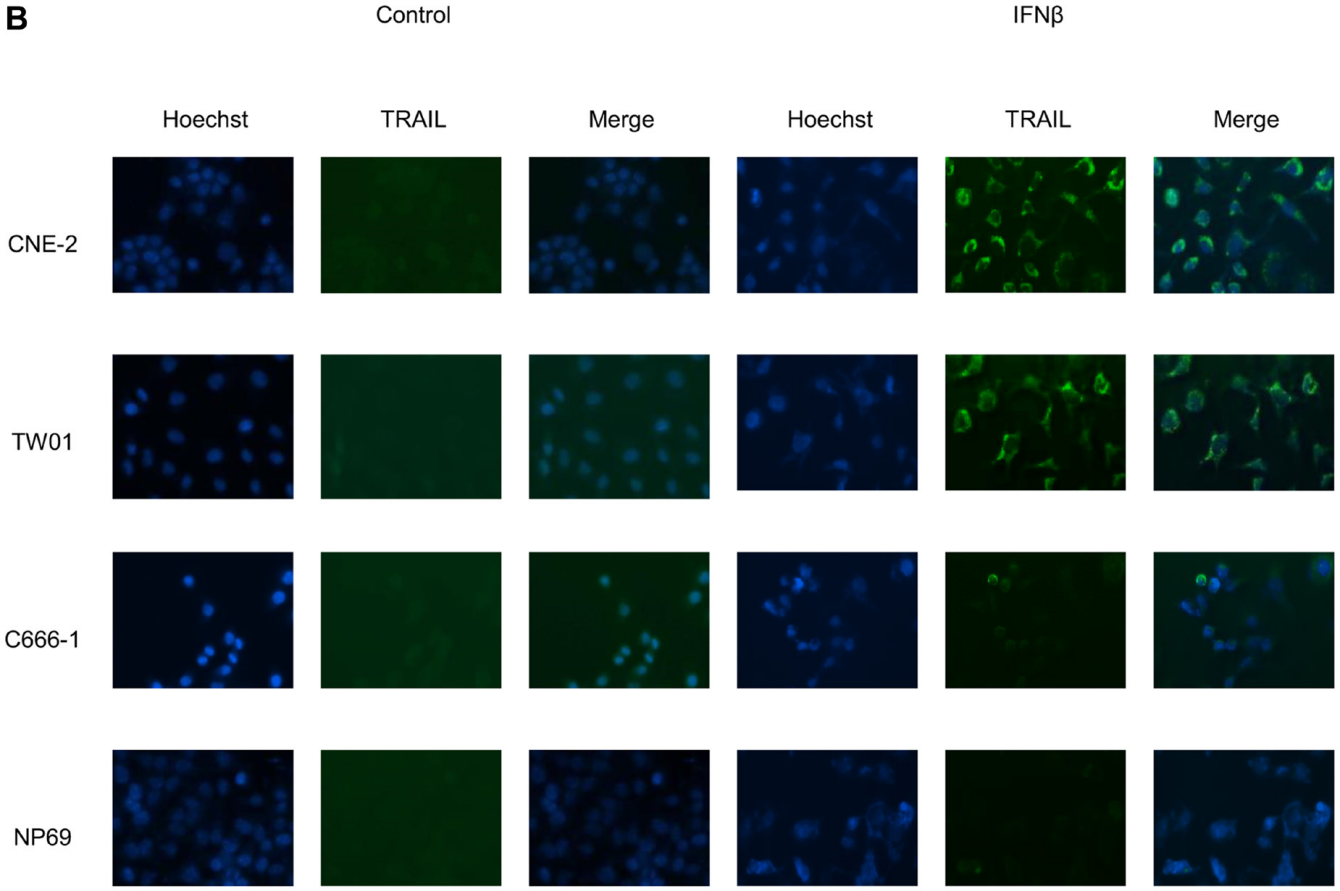
IFNβ induces surface expression of TRAIL in NPC cells. (**B**) Immunolocalization of TRAIL in NPC cells. Cells were treated for 72 h with IFNβ and stained for TRAIL as described in “Materials and Methods”. Nuclei were counterstained with Hoechst 33258. Immunofluorescence microscopy at 400× magnification demonstrates predominant localization of TRAIL on the cell surface.
