# Supplementary material for: Interferon beta induces apoptosis in nasopharyngeal carcinoma cells via the TRAIL-signaling pathway
Source: Oncotarget. 2018 Feb 12;9(18):14228–50. doi: 10.18632/oncotarget.24479 (PMC5865666; doi:10.18632/oncotarget.24479)
Supplement: Supplementary file 1 [file oncotarget-09-14228-s001.pdf]

Interferon beta induces apoptosis in nasopharyngeal carcinoma cells *via* the TRAIL-signaling pathway

SUPPLEMENTARY MATERIALS

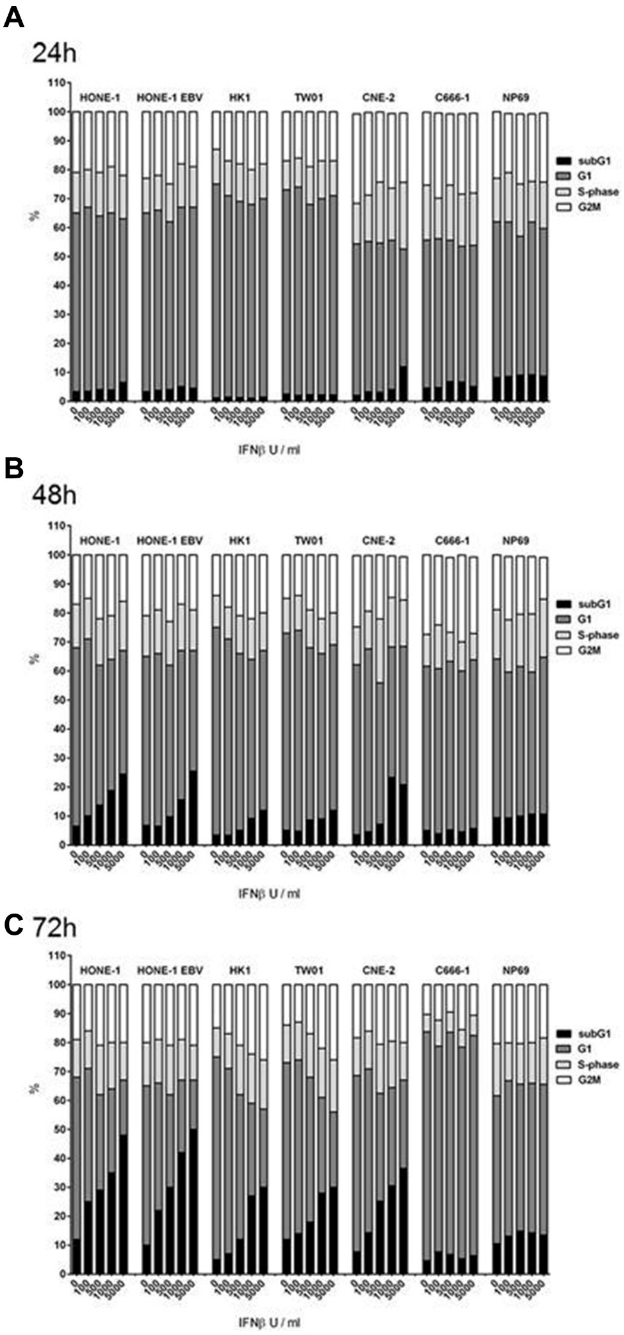

Supplementary Figure 1: Cell cycle distribution of NPC cells after treatment with IFNβ. NPC cell lines and the nasoepithelial cell line NP69 were treated with increasing concentrations of IFNβ for (**A**) 24 h, (**B**) 48 h, (**C**) 72 h, and cell cycle analysis was performed thereafter. No major effects of IFNβ on cell cycle distribution was seen; in cell lines HK1 and TW01 a slight dose-dependent increase in G2-phase was observed after 72 h of incubation with IFNβ. Data are presented as means ± S.E.M. of three independents experiments.

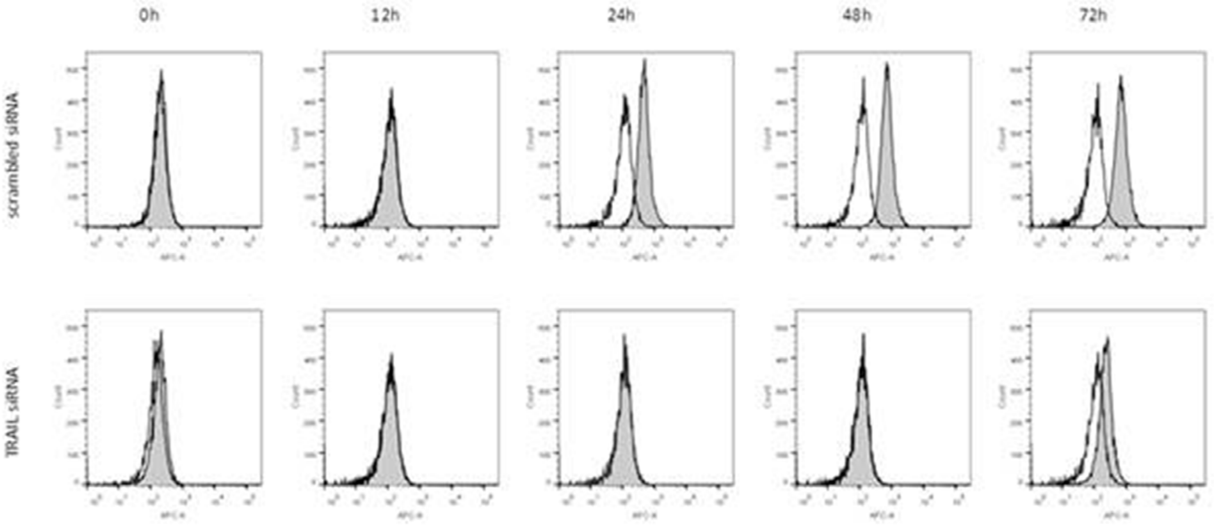

Supplementary Figure 2: Knockdown of TRAIL in NPC cells by small-interfering RNA (siRNA). Specific siRNA against TRAIL but not scrambled siRNA suppresses the expression of TRAIL in NPC cells. Cells of NPC cell line TW01 were incubated with 1,000 U/ml IFNβ and surface expression of TRAIL was analyzed by flow cytometry comparing IFNß-treated cells (gray area) against non-treated cells (white area).

	TH01	D21S11	D5S818	D13S317	D7S820	D16S539	vWA	TPOX	AMEL	% of Match
HELA	7	27, 28	11, 12	12, 13.3	8, 12	9, 10	16, 18	8, 12	X	
HONE-1	6, 7, 9	27, 30, 31	11, 12	10, 12, 13.3	10, 12	9, 10	14, 16	8, 12	X	65%
HONE-1 EBV	6, 7, 9	27, 30	11, 12	10, 12, 13.3	10, 12	9, 10, 11	14, 16	8, 9, 12	X	65%
CNE-2	7, 9	27, 30	11, 12	10, 12, 13.3	10	9, 10	14, 16	8, 9, 12	X	66%
HK1	7, 9.3	28, 29	12, 14	11	8, 11	10, 11	18, 19	8, 11	Y	41%
C666-1	6, 8	29, 31, 32	11	8, 11	11,12	11	17, 18	8, 11	Y	23%
TW01	6, 7, 9	26, 30	12, 13	10, 12, 13.3	10, 12	10, 11	14, 16	8, 12	X	47%
NP69	7	31	11	10, 12	11	11, 12	16, 19	11	Y	33%

Supplementary Figure 3: Short tandem repeated (STR) profiles of nasopharyngeal epithelial cell lines. STR profiles for NPC cell lines HONE-1, HONE-1 EBV and CNE-2 show 66%, 66% and 65% matching with HeLa cells, respectively, suggesting contamination. STR profiles for cell lines HK1, TW01, C666-1 and NP69 match only between 23% and 47% with HeLa cells, ruling out contamination
